# Data-Driven Uncertainty Quantification for Cardiac Electrophysiological Models: Impact of Physiological Variability on Action Potential and Spiral Wave Dynamics

**DOI:** 10.3389/fphys.2020.585400

**Published:** 2020-11-19

**Authors:** Pras Pathmanathan, Suran K. Galappaththige, Jonathan M. Cordeiro, Abouzar Kaboudian, Flavio H. Fenton, Richard A. Gray

**Affiliations:** ^1^U.S. Food and Drug Administration, Center for Devices and Radiological Health, Silver Spring, MD, United States; ^2^Department of Experimental Cardiology, Masonic Medical Research Institute, Utica, NY, United States; ^3^School of Physics, Georgia Institute of Technology, Atlanta, GA, United States

**Keywords:** uncertainty quantification, sensitivity analysis, variability, electrophysiology, correlation

## Abstract

Computational modeling of cardiac electrophysiology (EP) has recently transitioned from a scientific research tool to clinical applications. To ensure reliability of clinical or regulatory decisions made using cardiac EP models, it is vital to evaluate the uncertainty in model predictions. Model predictions are uncertain because there is typically substantial uncertainty in model input parameters, due to measurement error or natural variability. While there has been much recent uncertainty quantification (UQ) research for cardiac EP models, all previous work has been limited by either: (i) considering uncertainty in only a subset of the full set of parameters; and/or (ii) assigning arbitrary variation to parameters (e.g., ±10 or 50% around mean value) rather than basing the parameter uncertainty on experimental data. In our recent work we overcame the first limitation by performing UQ and sensitivity analysis using a novel canine action potential model, allowing all parameters to be uncertain, but with arbitrary variation. Here, we address the second limitation by extending our previous work to use data-driven estimates of parameter uncertainty. Overall, we estimated uncertainty due to population variability in all parameters in five currents active during repolarization: inward potassium rectifier, transient outward potassium, L-type calcium, rapidly and slowly activating delayed potassium rectifier; 25 parameters in total (all model parameters except fast sodium current parameters). A variety of methods was used to estimate the variability in these parameters. We then propagated the uncertainties through the model to determine their impact on predictions of action potential shape, action potential duration (APD) prolongation due to drug block, and spiral wave dynamics. Parameter uncertainty had a significant effect on model predictions, especially L-type calcium current parameters. Correlation between physiological parameters was determined to play a role in physiological realism of action potentials. Surprisingly, even model outputs that were relative differences, specifically drug-induced APD prolongation, were heavily impacted by the underlying uncertainty. This is the first data-driven end-to-end UQ analysis in cardiac EP accounting for uncertainty in the vast majority of parameters, including first in tissue, and demonstrates how future UQ could be used to ensure model-based decisions are robust to all underlying parameter uncertainties.

## 1. Introduction

Computational modeling of cardiac electrophysiology has long been used to generate hypotheses for experimental verification and investigate mechanisms underlying normal and pathological cardiac physiology. In recent years computational cardiac electrophysiological modeling has transitioned to clinical and regulatory applications. These include clinical trials that evaluate the ability of personalized whole-heart models to guide ablation therapy^1^, and the Comprehensive *in vitro* Pro-arrhythmia Assay (CiPA) program, which uses a computational model of the action potential (AP) as part of a framework for assessing drug cardiotoxicity (Strauss et al., 2018). These advances have coincided with a dramatic rise in the use of physics-based and physiological modeling in drug/device regulatory submissions supporting safety and efficacy/effectiveness of medical products, or in software within medical devices (Faris and Shuren, 2017; Morrison et al., 2018). In parallel, the medical devices community and other healthcare communities have focused attention on methods and best practices for ensuring the reliability of computational modeling approaches (ASME V&V 40, 2018).

Understanding the uncertainty in model predictions is acknowledged as a critical component of model credibility assessment (Oberkampf et al., 2004; National Research Council, 2012; ASME V&V 40, 2018). Just as measurement error is key to interpreting experimental measurements, supplementing computational model predictions with an estimate of uncertainty vastly improves the ability to make informed decisions. Uncertainty quantification (UQ) is the science of characterizing uncertainties in computational models. While this includes uncertainty in the model form, i.e., uncertainty in the best equations to model a system, the most common form of UQ involves two stages. First, characterizing the uncertainty in the model inputs—all the real-world measured quantities that are used in the model, such as model parameters, boundary conditions, and initial conditions. Second, “propagating” these uncertainties through the model to obtain the uncertainty in model outputs of interest. We refer to these two stages as uncertainty characterization and uncertainty propagation, respectively. With UQ inputs and outputs are generally represented using probability distributions, rather than taking fixed values.

For physiological models, the main reasons for input uncertainty are experimental measurement error and natural physiological variability. Both may be significant—typically orders of magnitude greater than measurement error and natural variability within engineering systems. Physiological models are typically non-linear, so the impact of input uncertainty on model outputs is difficult to predict without simulation. Both these challenges apply to cardiac electrophysiological modeling. Accordingly, there has been a lot of recent interest in UQ within the cardiac modeling community, and many groups are overcoming the challenges. For a detailed discussion on challenges and previous work see our previous discussion in Pathmanathan et al. (2019) (introduction), and a recent special edition devoted to cardiac model UQ (Mirams et al., 2020); also see the Discussion section in the present paper which will compare the approach used herein with other prominent approaches.

Cardiac action potential models are typically sets of ordinary differential equations (ODEs). Several hundred models for different species have been developed (Noble et al., 2012); it is not uncommon for a model to have dozens of equations governing dynamic behavior of transmembrane voltage, membrane gating variables and intra-cellular and extra-cellular ionic concentrations, altogether involving hundreds of parameters. For this reason, all previous cardiac AP model UQ has been limited in one or both of the following: (i) only considering uncertainty in a restricted subset of the parameters, for example considering ionic current maximum conductances as uncertain but keeping parameters for gating dynamics fixed; and (ii) using arbitrarily chosen variation in the inputs, for example letting a parameter vary in a range, such as ±10 or 50% around its mean value, rather than estimating true uncertainty in the input due to measurement error or physiological variability. In our first work on this topic (Pathmanathan et al., 2015), we estimated uncertainty due to physiological variability in two parameters characterizing steady-state inactivation of the fast sodium current, and propagated this through the model to assess its impact on the action potential. This was an end-to-end UQ analysis, but only considered one component (steady-steady inactivation) of just one ion channel, i.e., was heavily limited by (i)—uncertainty in the vast majority of parameters was neglected. In our previous paper (Pathmanathan et al., 2019), we developed a novel canine action potential model which included representations of six major currents (fast sodium, inward rectifier, transient outward, L-type calcium, rapidly and slowly activating delayed rectifier), but had just seven state variables and 33 parameters (excluding environmental parameters). This allowed us to overcome limitation (i) and perform a comprehensive analysis where we accounted for uncertainty in all 33 parameters, including all conductances, steady state activation/inactivation parameters, and time constant parameters. This provided a wealth of information on the general robustness of the model. However, it was still limited by (ii), because we prescribed arbitrary input variation.

In this paper we expand upon our previous work (Pathmanathan et al., 2019) by using experimental data to estimate the parameter uncertainty, and compute the impact of the parameter uncertainty on the action potential and spiral wave dynamics. Using our canine model developed in Pathmanathan et al. (2019), uncertainty due to population variability was estimated for all parameters in the five currents active during repolarization (inward rectifier, transient outward, L-type calcium, rapidly and slowly activating delayed rectifier currents); 25 parameters in all. We did not attempt to estimate uncertainty in fast sodium current parameters due to the known challenges in obtaining high quality experimental data on this current under physiological conditions which makes even estimating average values of parameters challenging (Berecki et al., 2010), let alone estimating population variability. We used a variety of methods for estimating population variability. Where relevant data was available, we estimated correlation between parameters across the population. Overall, we generated probabilistic representation of all 25 repolarization parameters in the model. We then propagated this uncertainty through the action potential model, to compute the impact on: (a) the action potential shape; (b) action potential duration (APD) prolongation following drug-induced partial block of the *I*_Kr_ current; and (c) spiral wave dynamics. We also investigated the role parameter correlation plays in governing AP shape. To our knowledge this is the first time such a data-driven end-to-end uncertainty quantification has been performed for more than a handful of AP model parameters, let alone for the vast majority, and the first such analysis in tissue. Our results provide for the first time information on the expected uncertainty in cardiac AP and spiral wave models given experimentally-derived parameter uncertainty in all repolarization parameters, and demonstrates how future UQ could be used to ensure cardiac model-based decisions are robust to all underlying parameter uncertainties.

## 2. Methods

### 2.1. Cell Model

The canine cell model of Pathmanathan et al. (2019) was used. In this model transmembrane voltage is governed by

(1) $$\begin{array}{l} C_{m}\frac{\text{d}V}{\text{d}t}+I_{\text{Na}}+I_{\text{K}1}+I_{\text{to}}+I_{\text{CaL}}+I_{\text{Kr}}+I_{\text{Ks}}=I_{\text{stim}}, \end{array}$$

where *V* is transmembrane voltage, $C_{m}=1\mu$F cm^−2^ is the specific membrane capacitance per unit area, and *I*_Na_, *I*_K1_, *I*_to_, *I*_CaL_, *I*_Kr_, and *I*_Ks_ are ionic currents (respectively: rapid sodium, inward rectifier, transient outward, L-type calcium, rapidly and slowly activating delayed rectifier), and *I*_stim_ is a stimulus current. The currents are formulated as

(2) $$\begin{array}{l} I_{\text{Na}}=g_{\text{Na}}m^{3}h^{2}\left(V-E_{\text{Na}}\right) \end{array}$$

(3) $$\begin{array}{l} I_{\text{K}1}=g_{\text{K}1}z_{\infty }\left(V-E_{\text{K}}\right) \end{array}$$

(4) $$\begin{array}{l} I_{\text{to}}=g_{\text{to}}r_{\infty }s\left(V-E_{\text{K}}\right) \end{array}$$

(5) $$\begin{array}{l} I_{\text{CaL}}=g_{\text{CaL}}d_{\infty }f\left(V-E_{\text{Ca}}\right) \end{array}$$

(6) $$\begin{array}{l} I_{\text{Kr}}=g_{\text{Kr}}x_{r}y_{\infty }\left(V-E_{\text{K}}\right) \end{array}$$

(7) $$\begin{array}{l} I_{\text{Ks}}=g_{\text{Ks}}x_{s}\left(V-E_{\text{K}}\right) \end{array}$$

where the *g*_X_ are ion channel maximal conductances, *m*, *h*, *z*_∞_(*V*), *r*_∞_(*V*), *s*, *d*_∞_(*V*), *f*, *x*_*r*_, *y*_∞_(*V*), and *x*_*s*_ are probability of gates being open [activation gates: *m*, *r*_∞_, *d*_∞_, *x*_*r*_, *x*_*s*_; inactivation gates: *h*, *z*_∞_(*V*), *s*, *f*, *y*_∞_(*V*)], and *E*_Na_, *E*_K_, *E*_Ca_ are the Nernst potentials for sodium, potassium, and calcium, respectively. Per Equations (2)–(7), gates *z*, *r*, *d*, and *y* are not state variables (not governed by an ODE); these gates are taken to instantaneously reach steady state. Gating variables, *m, h, s, f, x*_*r*_, *x*_*s*_ have dynamics governed by the ODE

$$\tau_{Y}\left(V\right)\frac{\text{d}Y}{\text{d}t}=Y_{\infty }\left(V\right)-Y$$

where *Y*_∞_ is the steady-state function and τ_*Y*_ is the time “constant.” Steady state functions for all gates are sigmoidal

$$Y_{\infty }\left(V\right)={\left(1+\exp\left(\frac{\pm \left(V-E_{Y}\right)}{k_{Y}}\right)\right)}^{-1}$$

with − for activation gates and + for inactivation gates, where *E*_*Y*_ is the half-activation/inactivation voltage for that gating variable and *k*_*Y*_ > 0 controls the slope of the sigmoid. For time constants, voltage dependence was not modeled except for the *h*-gate:

$$\tau_{Y}\left(V\right)=\{\begin{array}{cc} \frac{2\tau_{h0}\exp\left(\delta_{h}\left(V-E_{h}\right)/k_{h}\right)}{1+\exp\left(\left(V-E_{h}\right)/k_{h}\right)}, & \text{for }Y=h\\ \tau_{Y}^{*}, & \text{for }Y=m,s,f,x_{r},x_{s} \end{array}$$

where τ_*h*0_, δ_*h*_, $\tau_{m}^{*}$, $\tau_{s}^{*}$, $\tau_{f}^{*}$, $\tau_{xr}^{*}$, $\tau_{xs}^{*}$ are positive constants. Overall, this cell model has 33 parameters, excluding *E*_Na_, *E*_K_, and *E*_Ca_. In Pathmanathan et al. (2019), “nominal” values of each of these parameters were defined, and the impact of arbitrarily chosen variation was analyzed. In this present paper, we studied the impact of data-driven uncertainty in all *I*_K1_, *I*_to_, *I*_CaL_, *I*_Kr_, and *I*_Ks_ parameters (25 parameters in total). For the other parameters (eight *I*_Na_ parameters, and *E*_Na_, *E*_K_, and *E*_Ca_) nominal values from Pathmanathan et al. (2019) were used. A CellML version of this model [using mean values of repolarization parameters (see Table 1 in section 3) and nominal values of *I*_Na_ parameters and *E*_Na_, *E*_K_, and *E*_Ca_ (Pathmanathan et al., 2019)] is available upon request.

### 2.2. Uncertainty Characterization

For the 25 repolarization parameters, our aim was to derive a first-estimate of population variability based on available data. Since this the first time such a UQ analysis has been performed on this scale for cardiac models, we consider relatively crude estimates acceptable for some parameters, as long as they are derived from experimental data. We assumed the type of distribution (normal or log-normal) and estimated distribution scale parameters (e.g., means and variances). Parameters which are physiologically constrained to be positive (conductances *g*_*X*_, slopes *k*_*X*_, time constants, $\tau_{X}^{*}$) were assumed to follow log-normal distributions. The other parameters (half activation/inactivation voltages *E*_*X*_) were assumed to follow normal distributions.

Different types of data was available for the different repolarization currents, and accordingly a variety of different methods was used to characterize the parameter uncertainty. These are discussed in the following sections.

#### 2.2.1. *I*_K1_ Parameter Uncertainty

The inward rectifier current *I*_K1_ has three parameters: maximum conductance *g*_K1_, half-inactivation voltage *E*_*z*_, and slope of steady-state inactivation at half-inactivation voltage *k*_*z*_. Previously, in Pathmanathan et al. (2019), we determined values for the three *I*_K1_ parameters by fitting

(8) $$\begin{array}{l} F\left(g_{\text{K}1},E_{z}^{\text{exp}},k_{z},E_{K}^{\text{exp}}\right)=g_{\text{K}1}{\left(1+\exp\left(\frac{\left(V-E_{z}^{\text{exp}}\right)}{k_{z}}\right)\right)}^{-1}\left(V-E{\text{K}}^{\text{exp}}\right) \end{array}$$

to averaged voltage clamp recordings from canine epicardial cells (*n* = 12). Here, $E_{\text{K}}^{\text{exp}}$ represents unknown effective *E*_K_ in the experiments, which is dependent on the unknown intra and extracellular potassium ion concentrations. $E_{z}^{\text{exp}}$ represents the effective *E*_*z*_ in the experiments, and actual *E*_*z*_ was computed by shifting $E_{\text{z}}^{\text{exp}}$ to correspond to *E*_K_ = −85 mV, i.e., *E*_*z*_ = $E_{\text{z}}^{\text{exp}}-\left(E_{\text{K}}^{\text{exp}}+85\right)$. In essence, *E*_K_ and *E*_*z*_ were both fit and *E*_*z*_ shifted to correspond to *E*_K_ = −85 mV.

In the present paper, to calibrate with uncertainty, we fitted to recordings from the *n* = 12 individual cells. A “two-stage approach” was used. Stage 1 was to apply the above process using each cell's *I*-*V* curve (rather than the averaged data), to obtain parameters for each cell. Stage 2 was to fit a multivariate probability distribution to the set of (*g*_K1_, *E*_*z*_, *k*_*z*_) values. Allowing for possible correlation between parameters across the population, we assumed a multivariate normal distribution and computed the mean vector and covariance matrix from the 12 samples.

#### 2.2.2. *I*_to_ Parameter Uncertainty

The transient outward current *I*_to_ has six parameters: maximum conductance *g*_to_, half-activation voltage *E*_*r*_, activation slope *k*_*r*_, half-inactivation voltage *E*_*s*_, inactivation slope *k*_*s*_, and inactivation time constant $\tau_{s}^{*}$.

Previously, in Pathmanathan et al. (2019), nominal values for *g*_to_, *E*_*r*_ and *k*_*r*_ were determined by fitting

(9) $$\begin{array}{l} F\left(g_{\text{to}},E_{r},k_{r}\right)=g_{\text{to}}{\left(1+\exp\left(\frac{-\left(V-E_{r}\right)}{k_{r}}\right)\right)}^{-1}\left(V-E_{\text{K}}\right) \end{array}$$

to averaged voltage clamp recordings from canine epicardial cells (*n* = 16). *E*_K_ was not identifiable from this dataset so was set to be −85 mV. In the present paper, to calibrate with uncertainty, the two stage approach was used as described in section 2.2.1, which again allows for possible correlation between these parameters.

Inactivation parameters, *E*_*s*_ and *k*_*s*_ were determined by fitting *F*(*E*_*s*_, *k*_*s*_, α) = (1/(1 + exp((*V* − *E*_*s*_)/*k*_*s*_)) + α)/(1 + α) to voltage clamp recordings from canine epicardial cells (*n* = 14). The function is a sigmoid that decreases from 1 to α, and was chosen because the data exhibited an experimental artifact where peak current did not drop to zero at higher voltage. α was fit for each cell (to obtain more accurate *E*_*s*_, *k*_*s*_) but not used in the model. The two-stage approach was again used.

To assess variability in $\tau_{s}^{*}$, we first defined $\tau_{s}^{*}$ more precisely [than in our previous work (Pathmanathan et al., 2019)] to be the average value of voltage-dependent τ_*s*_(*V*) for voltages in the range 10–50 mV. This is consistent with the value of $\tau_{s}^{*}$ used in Pathmanathan et al. (2019), and is necessary to be able to meaningfully ask what the population variability in this quantity is (see Discussion in Pathmanathan et al., 2019). Using this definition, $\tau_{s}^{*}$ was computed for eight canine epicardial cells (raw data behind Figure 2 in Cordeiro et al., 2012). A log-normal distribution was assumed, and scale parameters μ, σ estimated from the eight samples.

#### 2.2.3. *I*_CaL_ Parameter Uncertainty

The *L*-type inward calcium current *I*_CaL_ has six parameters: maximum conductance *g*_CaL_, half-activation voltage *E*_*d*_, activation slope *k*_*d*_, half-inactivation voltage *E*_*f*_, inactivation slope *k*_*f*_, and inactivation time constant $\tau_{f}^{*}$.

In Pathmanathan et al. (2019), nominal values for *E*_*d*_, *k*_*d*_, *E*_*f*_, and *k*_*f*_, were taken directly from Iyer et al. (2012) (Table 2, normal, EPI). The same table in Iyer et al. (2012) reports means and standard errors for each of these values, so here we estimated uncertainty due to population variability by simply calculating standard deviations from the standard errors. *E*_*d*_ and *E*_*f*_ were assumed to be normally distributed with these means and standard deviations. *k*_*d*_ and *k*_*f*_ were assumed to be log-normally distributed, with parameters μ and σ^2^ that were computed by inverting the relationship between (mean,variance) and (μ, σ) for log-normal random variables. This provides relatively crude (compared to methods used for *I*_K1_ and *I*_to_) but data-driven estimates of uncertainty for these four parameters, although this approach provides no indication of any correlations between them.

In Pathmanathan et al. (2019), the nominal value of *g*_CaL_ was determined by finding the value of *g*_CaL_ such that (5) with *f* = 1 and *E*_*d*_, *k*_*d*_ at nominal values matched the experimental peak current of the *I*-*V* curve in Figure 3 (EPI) of Iyer et al. (2012) (using digitized data). Here, we first estimated experimental peak current standard deviation (*n* = 17 cells) from digitized peak current standard error in the same figure. We then determined (μ, σ) values for log-normally distributed *g*_CaL_ such that this variability in *g*_CaL_, together with the above variability in *E*_*d*_ and *k*_*d*_, when propagated through (5), gave rise to the experimental mean and standard deviation of peak current. This approach was only possible because the variability in *E*_*d*_ and *k*_*d*_ was not sufficient to explain the experimental variability in peak current. Specifically, peak current was estimated to be −3.90 ± 1.40 mS/cm^2^ (mean ± standard deviation) from Iyer et al. (2012) (Figure 3, EPI). With constant *g*_CaL_ = 0.115 (nominal value from Pathmanathan et al., 2019), propagating the above uncertainty in *E*_*d*_ and *k*_*d*_ through (5) leads to simulated peak current of −4.12 ± 0.41, less variability than in the experiments. Hence it is possible to match the experimental mean and standard deviation by introducing variability in *g*_CaL_.

In Pathmanathan et al. (2019), the nominal value of $\tau_{f}^{*}$ was chosen based on Figure 8 of Xiao et al. (2006). Here, we first re-defined $\tau_{f}^{*}$ as the average value of τ_*f*_(*V*) over voltages between 0 and 40 mV (to enable us to meaningfully ask what is the population variability in $\tau_{f}^{*}$). We then digitized the male and female means and standard errors in Figure 8 of Xiao et al. (2006), computed standard errors from standard errors, computed grouped (both sexes) means and standard deviation at each voltage, and then computed the mean and standard deviation of $\tau_{f}^{*}$ using the above definition.

#### 2.2.4. *I*_Kr_ Parameter Uncertainty

The rapidly activating delayed rectifier current *I*_Kr_ has six parameters: maximum conductance *g*_Kr_, half-activation voltage *E*_*xr*_, activation slope *k*_*xr*_, activation time constant $\tau_{xr}^{*}$, half-inactivation voltage *E*_*y*_, and inactivation slope *k*_*y*_.

In Pathmanathan et al. (2019) *E*_*xr*_, *k*_*xr*_, *E*_*y*_, and *E*_*y*_ were taken from Table 1 of Berecki et al. (2005). Here we used standard errors reported in that table to estimate uncertainty in these parameters, using the same method as described above for *I*_CaL_ parameters in section 2.2.3.

The parameters *g*_Kr_, $\tau_{xr}^{*}$, together with *I*_Ks_ conductance *g*_Ks_ (below), are fundamentally different to all other parameters in this model. In Pathmanathan et al. (2019) all other parameters had nominal values derived directly from voltage clamp data or data in the literature. These three parameters however were *calibrated* using the full action potential model, to match experimental restitution data. Therefore, an appropriate method for quantifying the variability in these parameters is not obvious. In principle, we could estimate population variability in the restitution curve, and determine probability distributions for *g*_Kr_, $\tau_{xr}^{*}$, and *g*_Ks_ that give rise to that variability in restitution. However, there are two problems with such an approach. First, it is inconsistent with our aim of studying the impact of parameter uncertainty on action potential characteristics, since AP characteristics would be used to determine the parameter uncertainty. More importantly, to be fully rigorous, the quantified uncertainty in all the other parameters should be accounted for (in the same way uncertainty in *E*_*d*_ and *k*_*d*_ was included when calibrating *g*_CaL_ to variability in peak *I*_CaL_ in section 2.2.3). But we already know from the results in Pathmanathan et al. (2019) that moderate levels of uncertainty lead to very wide ranging APDs and action potentials that do not repolarize, and therefore the uncertainty in simulated restitution curve due to variability in the other parameters, will be greater than the experimentally observed variability in restitution.

Therefore, we used the following approach. In Table 1 (see below), ${\hat{\sigma }}_{\text{LN}}$ is defined as the ratio of standard deviation to mean, for log-normally distributed parameters. Noting that the other conductances and time constants have ${\hat{\sigma }}_{\text{LN}}$ values of 28, 39, 13, 35, 31, and 22%, we set mean values for these three parameters to be their nominal value (from Pathmanathan et al., 2019) and chose standard deviations corresponding to ${\hat{\sigma }}_{\text{LN}}=40\%$, a conservatively wide-ranging distribution. Thus, these parameters have distributions that are not arbitrarily chosen, but only based on information regarding other parameters.

#### 2.2.5. *I*_Ks_ Parameter Uncertainty

The slowly activating delayed rectifier current *I*_Ks_ has four parameters: maximum conductance *g*_Ks_, half-activation voltage *E*_*xs*_, activation slope *k*_*xs*_, and activation time constant $\tau_{xs}^{*}$. In Pathmanathan et al. (2019) nominal values of *E*_*xs*_, *k*_*xs*_, and $\tau_{xs}^{*}$ uncertainty were taken from Liu and Antzelevitch (1995) (text); here we set mean values as the nominal values and computed standard deviations from reported standard errors using values in Liu and Antzelevitch (1995). For *g*_Ks_, see section 2.2.4.

#### 2.2.6. Comparing Parameter Uncertainty

Once uncertainty in model parameters has been quantified, and before the impact of that uncertainty on model outputs was investigated, we wished to directly compare the uncertainty in parameters. To do so, we introduced two relative measures of uncertainty. For half activation/inactivation voltages (normally-distributed), we defined ${\hat{\sigma }}_{\text{N}}$ as standard deviation divided by a representative range of 100mV. For all other parameters (all constrained to be positive, all log-normally distributed), we defined ${\hat{\sigma }}_{\text{LN}}$ as standard deviation divided by mean. Both ${\hat{\sigma }}_{\text{N}}$ and ${\hat{\sigma }}_{\text{LN}}$ are analogous to $\hat{\sigma }$ in Pathmanathan et al. (2019).

### 2.3. Single Cell and Tissue Simulations

Single cell simulations were run using Chaste, a general purpose package for physiological simulations (Mirams et al., 2013). ODEs were solved in Chaste using the CVODE adaptive ODE solver (Hindmarsh et al., 2005). Initial conditions were dependent on parameter values (see Pathmanathan et al., 2019). Electrical activity in tissue was simulated using the monodomain equations

$$\chi \left(C_{m}\frac{\partial V}{\partial t}+I_{\text{Na}}+I_{\text{K}1}+I_{\text{to}}+I_{\text{Kr}}+I_{\text{Ks}}+I_{\text{CaL}}\right)=\nabla \cdot \left(\sigma \nabla V\right)$$

coupled to (2)–(7), where χ = 1, 400cm^−1^ is the surface-area-to-volume ratio, and σ = 1.4 mS/cm is the bulk conductivity. Tissue simulations were performed with a GPU-based finite difference solver implemented in WebGL 2.0 using the Abubu.js library (Kaboudian et al., 2019a,b). The WebGL 2.0 implementation of cardiac models provide significant speedup and better performance compared to other GPU implementations, such as NVIDIA CUDA or OpenACC (Kaboudian et al., 2019c). Simulations were performed on a 16 by 16 cm square domain, with initial conditions that were the same as single cell simulations except a gradient in *V* was prescribed in the *x*-direction (linear, −85 mV to −5 mV) and a gradient in *h* was prescribed in the *y*-direction (linear, ranging from *h* = 0.1 to 0.6). With these initial conditions a spiral wave spontaneously forms at *t* = 0, consistently across the majority of the parameter values used. We quantified the impact of the parameter uncertainty on two quantities of interest: (i) number of phase singularities [PSs; centers of reentrant waves, computed as described previously (Pathmanathan and Gray, 2015; Galappaththige et al., 2019)] at times *t* = 1, 000, 1, 500, and 2, 000 ms; and (ii) for the node at the center of the domain, average cycle length for all full beats in the window *t* = 1, 000 to *t* = 2, 000 ms. (Note that this is an average over time for a single parameter set, not an average over parameter space).

### 2.4. Uncertainty Propagation, Sensitivity Analysis, Non-behavioral Analysis

The same analytic methods as in our previous paper were used; see Pathmanathan et al. (2019) for a full description. Briefly, simple Monte Carlo sampling was used for uncertainty propagation. For single cell simulations we used *N* = 100, 000 and for tissue simulations we used *N* = 1, 000 due to increased computational cost. All simulation results are used for computing histograms, but when plotting representative simulations we plotted 1,000 random selected action potential traces for single cell simulations and 10 for tissue, and 100 randomly selected snapshots of activity in tissue. For global sensitivity analysis (determining how much the uncertainty in an output can be apportioned to the underlying uncertainties in each input), variance-based sensitivity indices were computed using the Saltelli sampling method and the SALib library (Herman and Usher, 2017) (*N* = 10, 000 per uncertain parameter). Non-behavioral analysis was used to identify which parameters were responsible for different classes of behavior observed (e.g., determining which parameters where highly influential in AP repolarization failure, when this occurred). Cumulative distribution functions (CDFs) of “parameter value given behavior occurred” and “parameter value given behavior did not occur” were computed and the Kolmogorov-Smirnov test was used to test if the CDFs were statistically different, which indicates that the parameter is influential in whether the behavior occurs or not. Highly influential parameters were defined as those for which the test statistic was >0.2.

## 3. Results

### 3.1. Uncertainty in Parameters

**Figure 1:**
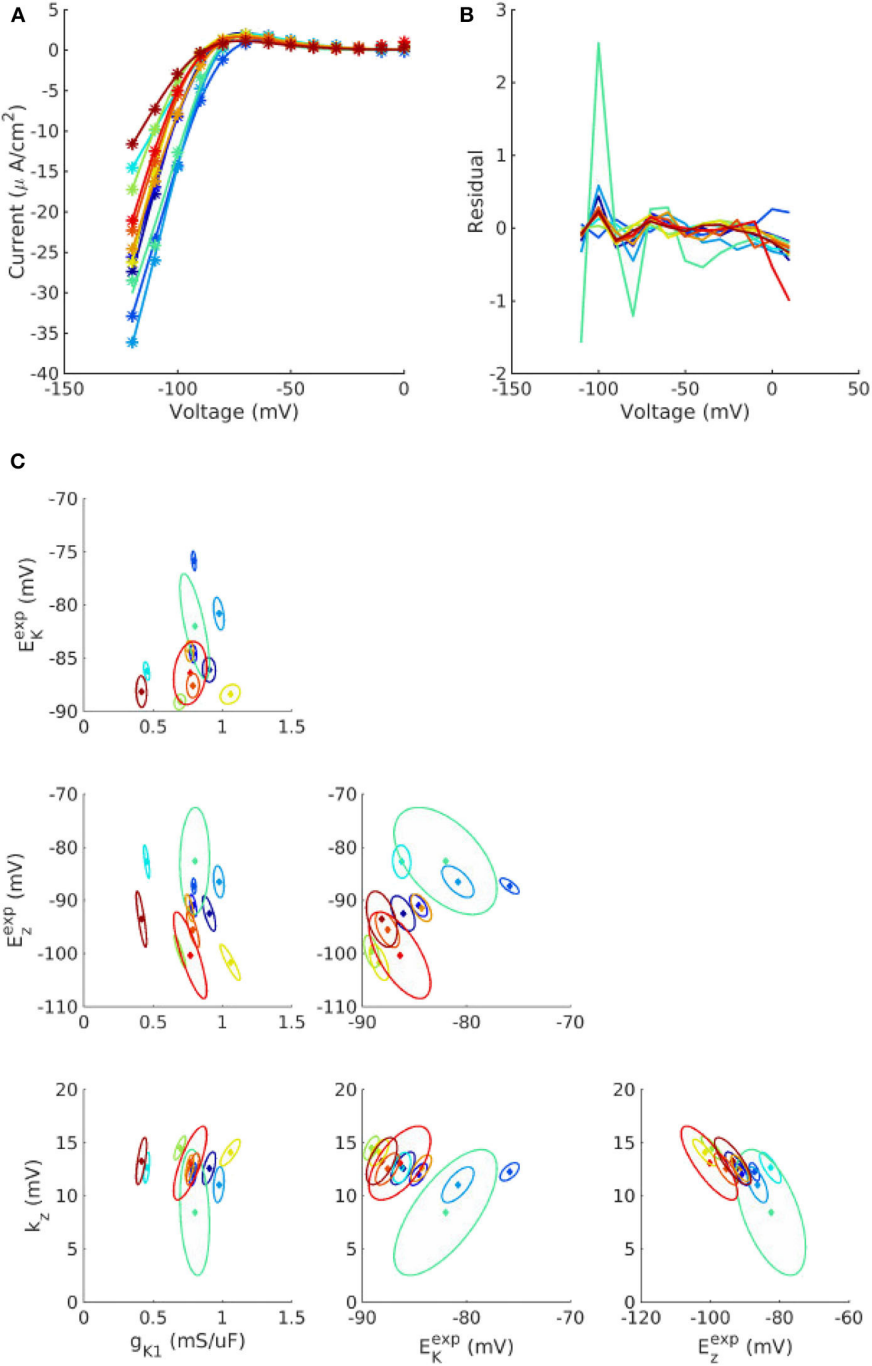
Individualized fits of *I*_K1_ parameters. **(A)** Experiment voltage clamp data (stars) and fitted model (line) for *n* = 12 cells. **(B)** Corresponding residuals (difference between experiment and fitted model). **(C)** Cell-specific parameters (solid diamonds) and cell-specific 90% confidence regions (ellipses) for the four fitted parameters, *g*_K1_, $E_{z}^{exp}$, *k*_*z*_, and $E_{K}^{exp}$, for each of the cells. See text for definitions of parameters.

Figure 1 plots the individualized fits of (8) to *I*_K1_ voltage clamp data recorded from 12 cells. Each color represents a different cell. Each point in parameter space represents the fitted values for that cell. Also plotted as ellipses are 90% confidence regions for each fitted parameter, using the variance-covariance matrix $\hat{\sigma }{\left(J^{T}T\right)}^{-1}$, where $\hat{\sigma }$ is the estimated residual variance and *J* is the Jacobian matrix with entries $J_{j}=\frac{\partial f}{\partial p_{j}}$. For most cells the calibration uncertainty (the ellipses) is small, but somewhat surprisingly for a few cells the calibration uncertainty was similar in size to the population variability across the cells (range of points). Note that confidence regions are presented here for illustration and not included in subsequent analysis. See discussion of limitations in section 4. See Supplementary Figures 1, 2, for the corresponding results for *I*_to_ activation and inactivation parameters. Some correlation was observed between *I*_K1_ parameters, between *I*_to_ activation parameters, and to a lesser extent between *I*_to_ inactivation parameters (see trends in fitted parameter values in Figure 1C, Supplementary Figures 1, 2).

The final probability distributions that were derived for each parameter are provided in the Appendix, and summarized in Table 1. The non-zero off-diagonal elements of the covariance matrices for *I*_K1_ and *I*_to_ parameters (Appendix) indicate the observed correlation. Looking first at ${\hat{\sigma }}_{\text{N}}$, it is remarkable that all half-activation/inactivation values except *E*_*xs*_ have similar levels of uncertainty: each has ${\hat{\sigma }}_{\text{N}}$ in the 4–8% range. This is despite a variety of methods and data sources being used to obtain these values. Uncertainty in *E*_*xs*_ is much larger $({\hat{\sigma }}_{\text{N}}=34\%$). This value was derived directly from the large standard error for *E*_*xs*_ reported in Liu and Antzelevitch (1995), and potentially dominated by measurement error rather than true variability. Although the experimental data on *I*_Ks_ activation in canine epicardial cells are sparse, a more recent study (Obreztchikova et al., 2006) exhibited variation less than (Liu and Antzelevitch, 1995) and more comparable with the other parameters. Uncertainty in the five conductances *g*_*X*_ was in the ${\hat{\sigma }}_{\text{LN}}=$ 28–40% range (two chosen to be 40%). Different data and methods were used to determine the variability of *g*_K1_, *g*_to_, and *g*_CaL_; it is interesting that the results are similar in magnitude. There was less uncertainty in slopes *k*_*X*_, all but one around 15%. Time constant uncertainty was in the 20–40% range. All values are considerably higher than the 1, 3, and 5% we prescribed in our previous work (Pathmanathan et al., 2019).

**Table 1:** Estimated mean and standard deviations for all repolarization parameters.

**Current**	**Param**	Distribution^*^	**Co-variates^†^**	**Mean**	**Std dev**	**${\hat{\sigma }}_{\text{N}}$ (%)**	**${\hat{\sigma }}_{LN}$ (%)**
*I*_K1_	*g*_K1_	Lognormal	*E*_*z*_, *k*_*z*_	0.772	0.217		28
	*E*_*z*_	Normal	*g*_K1_, *k*_*z*_	−92.1	5.01	5	
	*k*_*z*_	Lognormal	*g*_K1_, *E*_*z*_	12.4	1.73		14
*I*_to_	*g*_to_	Lognormal	*E*_*r*_, *k*_*r*_	0.172	0.0676		39
	*E*_*r*_	Normal	*g*_to_, *k*_*r*_	14.4	5.78	6	
	*k*_*r*_	Lognormal	*g*_to_, *E*_*r*_	11.8	1.63		14
	*E*_*s*_	Normal	*k*_*s*_	−48.1	6.12	6	
	*k*_*s*_	Lognormal	*E*_*s*_	3.29	0.755		23
	$\tau_{s}^{*}$	Lognormal		9.91	1.28		13
*I*_CaL_	*g*_CaL_	Lognormal		0.108	0.0378		35
	*E*_*d*_	Normal		0.7	3.71	4	
	*k*_*d*_	Lognormal		4.3	0.742		17
	*E*_*f*_	Normal		−15.7	7.01	7	
	*k*_*f*_	Lognormal		4.6	0.62		13
	$\tau_{f}^{*}$	Lognormal		30.4	9.45		31
*I*_Kr_	*g*_Kr_	Lognormal		0.056	0.0224^‡^		40^‡^
	*E*_*xr*_	Normal		−26.6	4.43	4	
	*k*_*xr*_	Lognormal		6.5	0.949		15
	$\tau_{xr}^{*}$	Lognormal		334	133.6^‡^		40^‡^
	*E*_*y*_	Normal		−49.6	7.8	8	
	*k*_*y*_	Lognormal		23.5	1.5		6
*I*_Ks_	*g*_Ks_	Lognormal		0.008	0.0032^‡^		40^‡^
	*E*_*xs*_	Normal		24.6	33.94	34	
	*k*_*xs*_	Lognormal		12.1	1.70		14
	$\tau_{xs}^{*}$	Lognormal		628	140		22

See Appendix for actual distributions. Units: g_X_ in mS/cm^2^, E_X_ in mV, k_X_ mV, $\tau_{X}^{*}$ in ms.

* *Type of distributions was prescribed, not inferred*.

† *Co-variates based on availability of data, not inferred*.

‡ *Standard deviation chosen so ${\hat{\sigma }}_{\text{LN}}=40\%$; see text. ${\hat{\sigma }}_{N}$ and ${\hat{\sigma }}_{\text{LN}}$ are relative measures of uncertainty; see text for precise definitions. INa parameters and EK, ENa, ECa were fixed at nominal values from Pathmanathan et al. (2019)*.

### 3.2. Impact of Parameter Uncertainty on Action Potential

**Figure 2:**
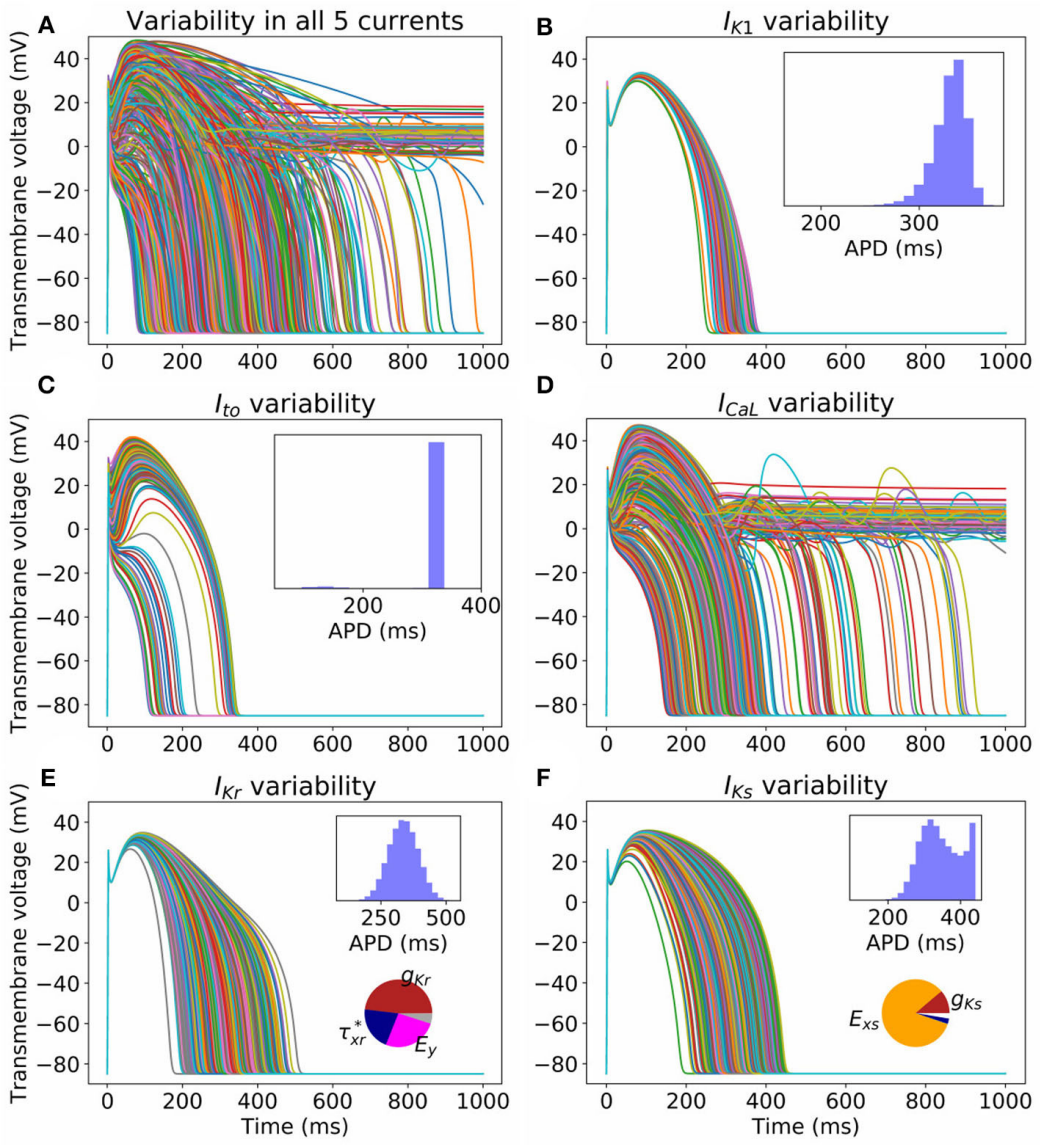
Impact on action potential of uncertainty (due to population variability, derived from experimental data) in all **(A)** or individual currents **(B–F)**. Each figure displays 1,000 sample action potentials, together with APD histograms (*N* = 100, 000). Pie charts show first-order Sobol sensitivity indices.

Figure 2 illustrates the impact of the quantified parameter uncertainty on the predicted action potential. Figure 2A plots 1,000 sample APs, with all 25 parameters sampled from the probability distributions provided in the Appendix. Unsurprisingly, given the wide range of APs observed in our previous work with just $\hat{\sigma }=5\%$, a very wide range of APs was observed. Figures 2B–F plot sample APs with all parameters in each current uncertain but all other parameters fixed at mean values, with APD histograms (*N* = 100, 000, requiring about 45s of computational time on a desktop using 20 CPUs) and sensitivity indices in insets. Sensitivity indices were not computed for the *I*_K1_ and *I*_to_ results because for these currents there is parameter correlation, and methodology for calculating sensitivity indices given correlated parameters is not well-established (Most, 2012). The uncertainty in *I*_CaL_ parameters has the greatest impact on the action potential, with early-after depolarizations (EADs) and repolarization failure occurring in some cases. No such behavior is observed for any other current. Uncertainty in *I*_Kr_ and *I*_Ks_ significantly affects APD. For *I*_Ks_ the sensitivity analysis shows that this is almost entirely due to the uncertainty in *E*_*xs*_, which as discussed in section 3.1 is most likely dominated by measurement error. Surprisingly, the distribution of APD in the *I*_Ks_ results is bi-modal. To investigate why, we plotted APD as a function of *E*_*xs*_, both with other parameters held fixed and with other parameters varying (see Supplementary Figure 3). The figure shows that *E*_*xs*_ takes values in the range −75 to 150 mV (perhaps wider-ranging than that which occurs in reality as discussed above). There is a non-linear relationship between APD and *E*_*xs*_, but at greater values of *E*_*xs*_ (e.g., >75 mV) the relationship is nearly flat (APD always near 450 ms), which is expected because at these values of *E*_*xs*_ *I*_Ks_ will not activate and therefore changes in *E*_*xs*_ will not impact APD. This explains the clustering of APD values near this value, hence the second peak in the histogram in Figure 2F.

Uncertainty in *I*_K1_ parameters has the least impact on AP shape. Uncertainty in *I*_to_ parameters has significant effect on phase 1 of the AP, but with large probability (*p* = 0.97) does not heavily affect APD. There is a small probability (*p* = 0.03) of AP shortening (APD < 200 ms).

Non-behavioral analysis (see section 2.4) was performed to determine parameters responsible for different behaviors. *I*_CaL_ APs (*N* = 100, 000) were generated and categorized as being either: normal; exhibiting loss of dome morphology; exhibiting EADs; or repolarization failure. *I*_to_ APs were categorized as either being normal or exhibiting reduced AP, delineated based on whether the AP exhibited spike-and-dome morphology or not (see Figure 2C). The results are provided in Table 2.

**Table 2:** Results from non-behavioral analysis to determine which parameters are highly influential in different behaviors that occur in simulated action potentials under *I*_CaL_ and *I*_to_ parameter uncertainty.

**Current of interest**	**Behavior observed**	**Influential parameters**
*I*_CaL_	Loss of dome	*g*_CaL_, *E*_*d*_, $\tau_{f}^{*}$
	Early-after depolarizations	*E*_*d*_, *E*_*f*_
	Repolarization failure	*g*_CaL_, *E*_*d*_, *E*_*f*_
*I*_to_	Action potential shortening	*g*_to_, *E*_*r*_, *k*_*r*_, $\tau_{s}^{*}$

### 3.3. Impact of Parameter Correlation

**Figure 3:**
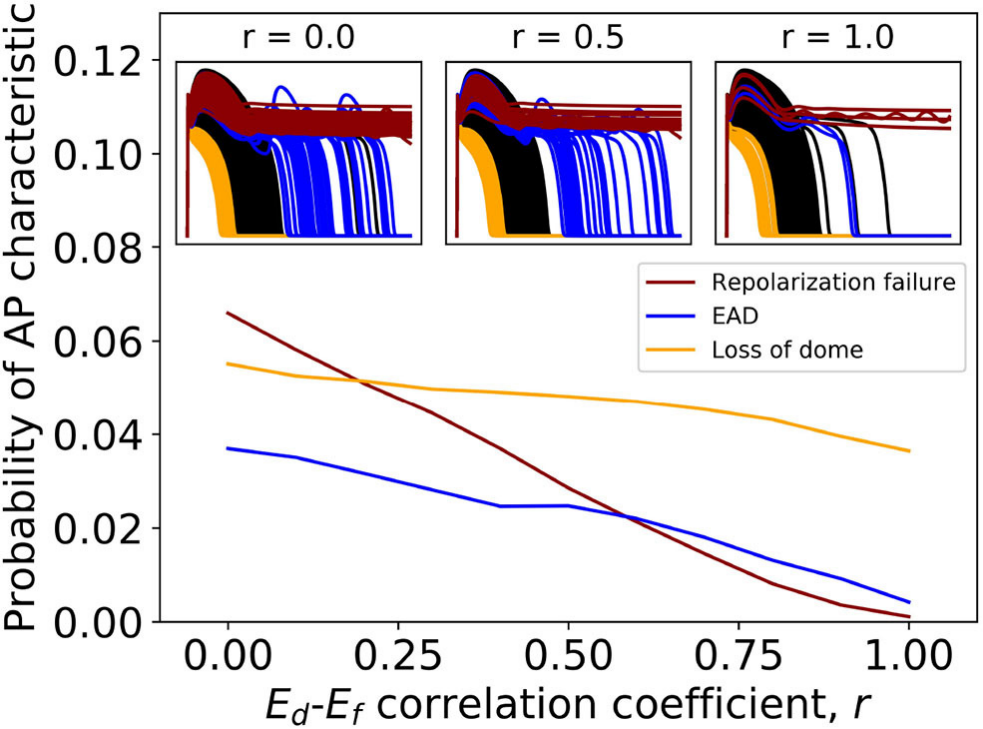
Effect of *introducing* correlation between half-activation voltage *E*_*d*_ and half-inactivation voltage *E*_*f*_ on *I*_CaL_ variability results. Probabilities of repolarization failure, early-after depolarizations (EADs) and loss of dome morphology all decreased with increasing correlation.

Table 2 shows that variability in *E*_*d*_ and *E*_*f*_ (steady state half-activation and half-inactivation voltages for the *d* and *f* gates in the *I*_CaL_ current) are influential in determining EADs or repolarization failure. Previously in Pathmanathan et al. (2019), we came to the same conclusion using arbitrary variation, and we hypothesized that these parameters may be correlated in reality. The probability distributions derived for *E*_*d*_ and *E*_*f*_ (see Appendix) assume no correlation, because we had no data allowing us to infer any correlation (section 2.2.3). In other words, the covariance matrix for (*E*_*d*_, *E*_*f*_) is diagonal. To assess if missing parameter correlation could be responsible for the wide range of APs observed under *I*_CaL_ uncertainty, we introduced correlation between *E*_*d*_ and *E*_*f*_ at a range of different levels, and computed the probability of loss of dome morphology, EADs, or repolarization failure. Different values of correlation coefficient *r* were chosen, spanning *r* = 0 (no correlation, i.e., above results) to *r* = 1 (perfect correlation). Off-diagonal elements of the (*E*_*d*_, *E*_*f*_) covariance matrix were set to covariance values corresponding to the chosen *r*. The results are plotted in Figure 3. All behaviors became less likely with increasing *E*_*d*_-*E*_*f*_ correlation, and the probability of EADs or repolarization failure approaches zero at perfect correlation. EADs and repolarization failure are likely related to the *I*_CaL_ window current, which is directly impacted by the values of *E*_*d*_ and *E*_*f*_. This raises the question of whether correlation between *E*_*d*_ and *E*_*f*_ impacts the magnitude of the window current, or location, or both. We computed the distribution of window current magnitude and location, for different values of *r*, and determined that correlation does *not* affect *I*_CaL_ window current location but it does affect window current magnitude (see Supplementary Figure 4). Specifically, correlation between *E*_*d*_ and *E*_*f*_ reduces the probability of the window current having larger magnitude, and through this reduces the probability of EADs.

**Figure 4:**
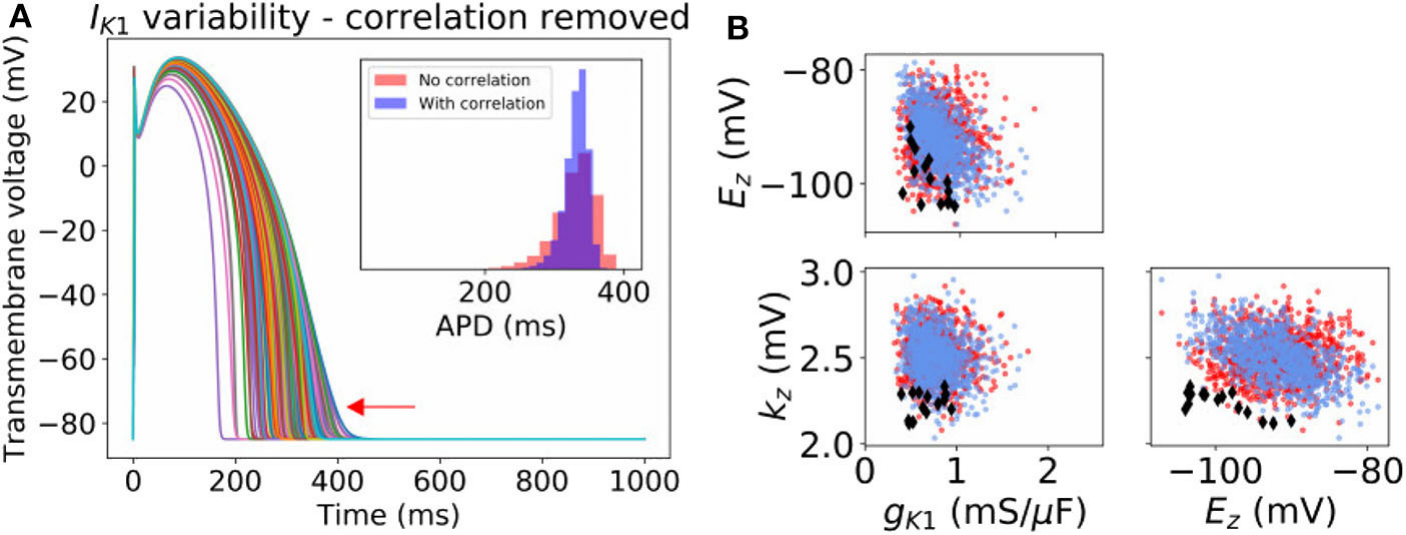
Effect of neglecting inferred correlations in *I*_K1_ parameters. **(A)** 1,000 sample action potentials with histograms of APD (*N* = 100, 000) in inset. Red arrow indicates some non-physiological behavior that arises upon neglecting the correlation between *I*_K1_ parameters. **(B)** Corresponding sampled parameters *g*_K1_, *E*_*z*_, *k*_*z*_. Blue dots: sampling from original distribution which included parameter correlation. Red dots *and* black diamonds: samples from distribution where correlation was removed. Black diamonds: parameter values which led to non-physiological action potentials.

For *I*_K1_ and *I*_to_, our probability distributions include parameter correlation (see covariance matrices in Appendix). To further investigate the importance of parameter correlation, we removed these correlations (i.e., made the covariance matrices diagonal), and re-computed APs. Results are shown in Figure 4A for *I*_K1_ and Supplementary Figure 5 for *I*_to_. Loss of correlation led to slightly wider-ranging APs; interestingly loss of *I*_K1_ parameter correlation led to some non-physiological APs (see arrow in Figure 4A). To understand why, we visualized the parameters values that led to the non-physiological APs in parameter space (see Figure 4B). The non-physiological APs were associated with the region of parameter space where *E*_*z*_ and *k*_*z*_ both took low values, and additionally *g*_K1_ was in the lower half of its set of values. In the original probability distribution, before correlation was removed, *E*_*z*_ and *k*_*z*_ were somewhat negatively correlated, which meant there was very small probability of sampling the region where *E*_*z*_ and *k*_*z*_ both took low values. When correlation was removed, the probability of sampling this region became non-negligible. *E*_*z*_ and *k*_*z*_ both taking low values, together with smaller *g*_K1_, corresponds to a small or near-zero *I*_K1_ current which gives rise to the non-physiological APs.

Overall, both correlation experiments (Figures 3, 4) suggest parameter correlation could play an important role in ensuring simulated APs are physiological when parameter variability is modeled.

### 3.4. Impact of Parameter Uncertainty on Drug-Induced Action Potential Duration Prolongation

**Figure 5:**
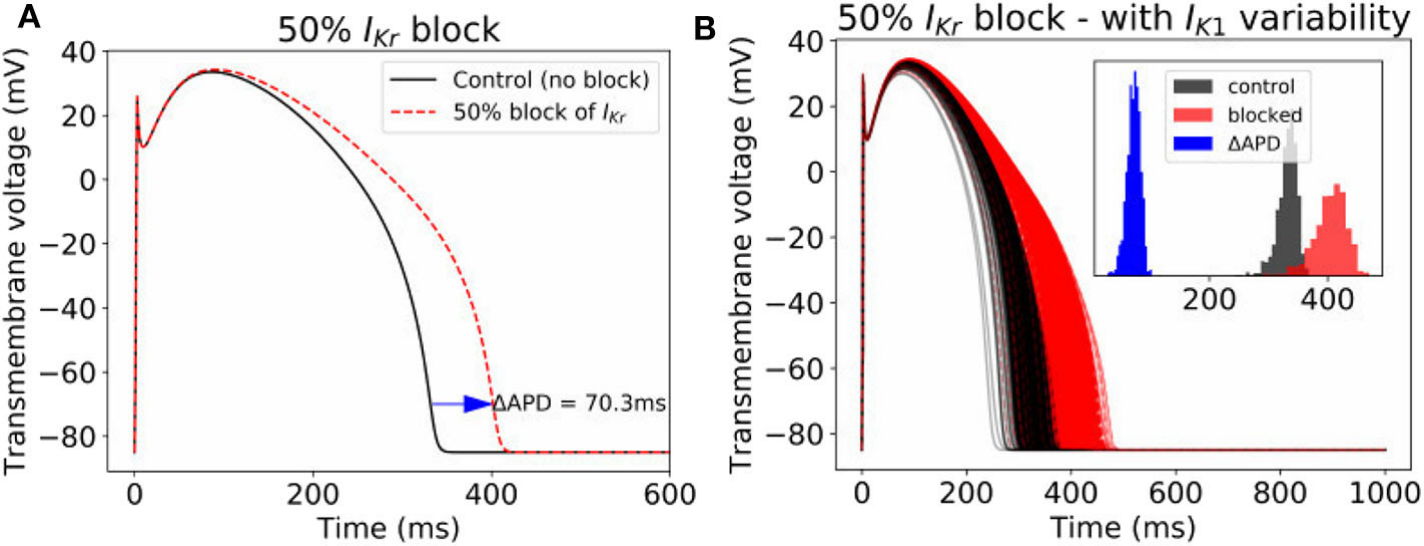
Impact of parameter variability on predictions of APD prolongation under 50% block of *I*_Kr_. **(A)** APD prolongation under 50% *I*_Kr_ block, with no parameter variability. **(B)** corresponding action potentials (1,000 samples) when *I*_K1_ variability is included, with histograms of the control APs, blocked APs, and ΔAPD in inset.

**Figure 6:**
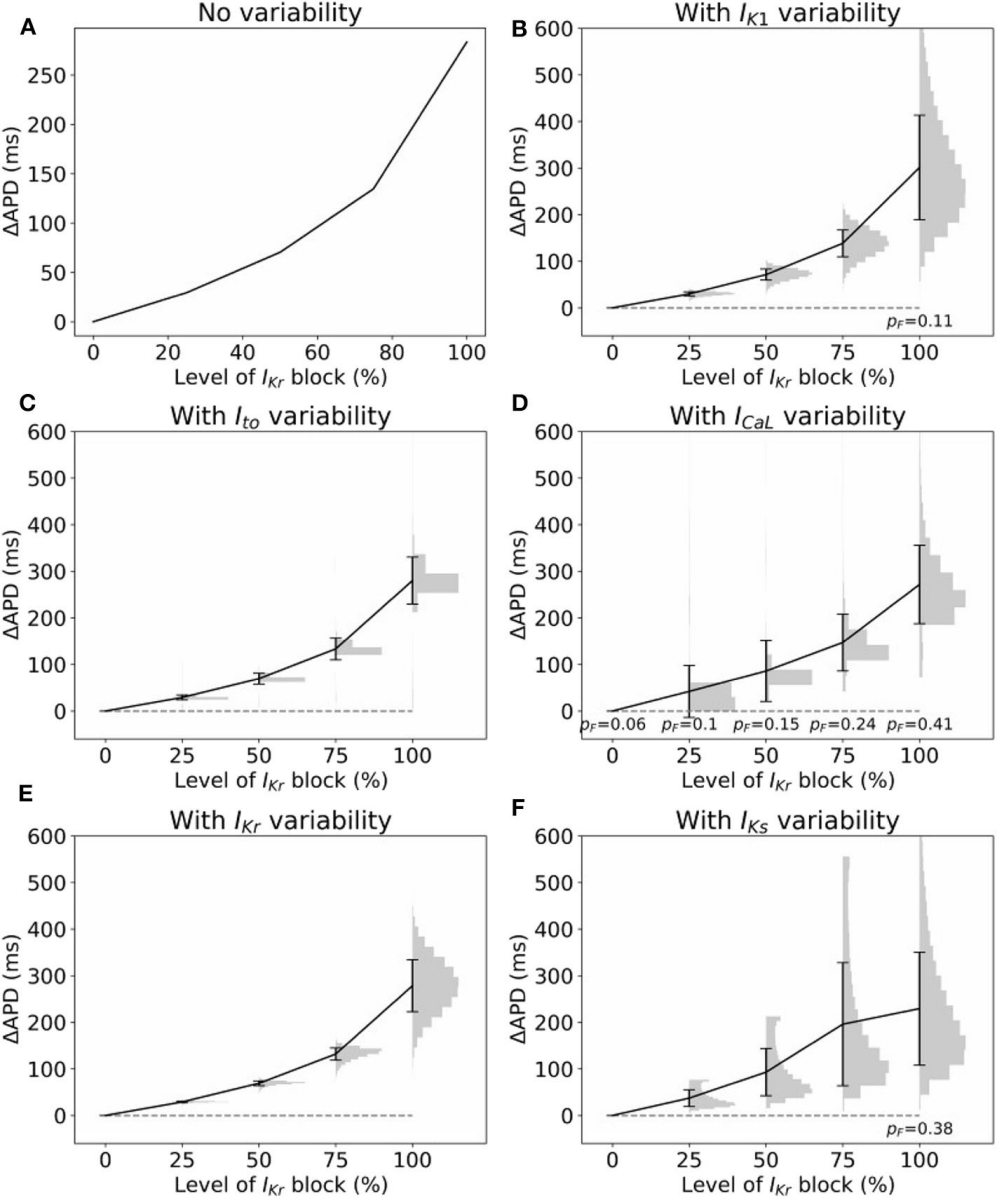
Impact of parameter variability on predictions of APD prolongation under block of *I*_Kr_. **(A)** APD prolongation as a function of *I*_Kr_ block, with no variability accounted for. **(B–F)** Corresponding results considered variability in specific current, with parameters in other currents held fixed. Black lines are means, error bars are ±1 standard deviation, histograms show distribution of (ΔAPD | APD computable). In some cases APD was not computable (“failure”), because either control or block AP did not repolarize. *p*_*F*_ represents probability of such failures. All histograms use 20 bins.

Often, cardiac models are not used to predict absolute quantities. Many times users are only interested in relative differences, for example changes in simulation results under drug block or when a proposed therapy is applied. While we observed large variability in predicted APs in Figure 2, we hypothesized that relative differences would be much more robust (less variable) to the underlying parameter uncertainty. To test this hypothesis, we computed the uncertainty in APD prolongation to partial block of the *I*_Kr_ current. Figure 5A illustrates the control action potential (no block) and the action potential under 50% block of *I*_Kr_, without including impact of parameter uncertainty. APD prolongation was 70.3 ms. Figure 5B then demonstrates the impact of *I*_K1_ variability (only). The control uncertainty is the same as shown in Figure 2B. Surprisingly, ΔAPD, a relative quantity, is heavily affected by underlying uncertainty, and its total uncertainty is similar in magnitude to the control uncertainty. This is because the APDs under partial *I*_Kr_ block (red traces/histogram) are very wide ranging. Figure 6 repeats this analysis for the full range of *I*_Kr_ block (0–100%), and with variability in all five currents. Parameter uncertainty always has a significant affect on results, especially at greater levels of block.

### 3.5. Impact of Parameter Uncertainty on Spiral Wave Dynamics

**Figure 7:**
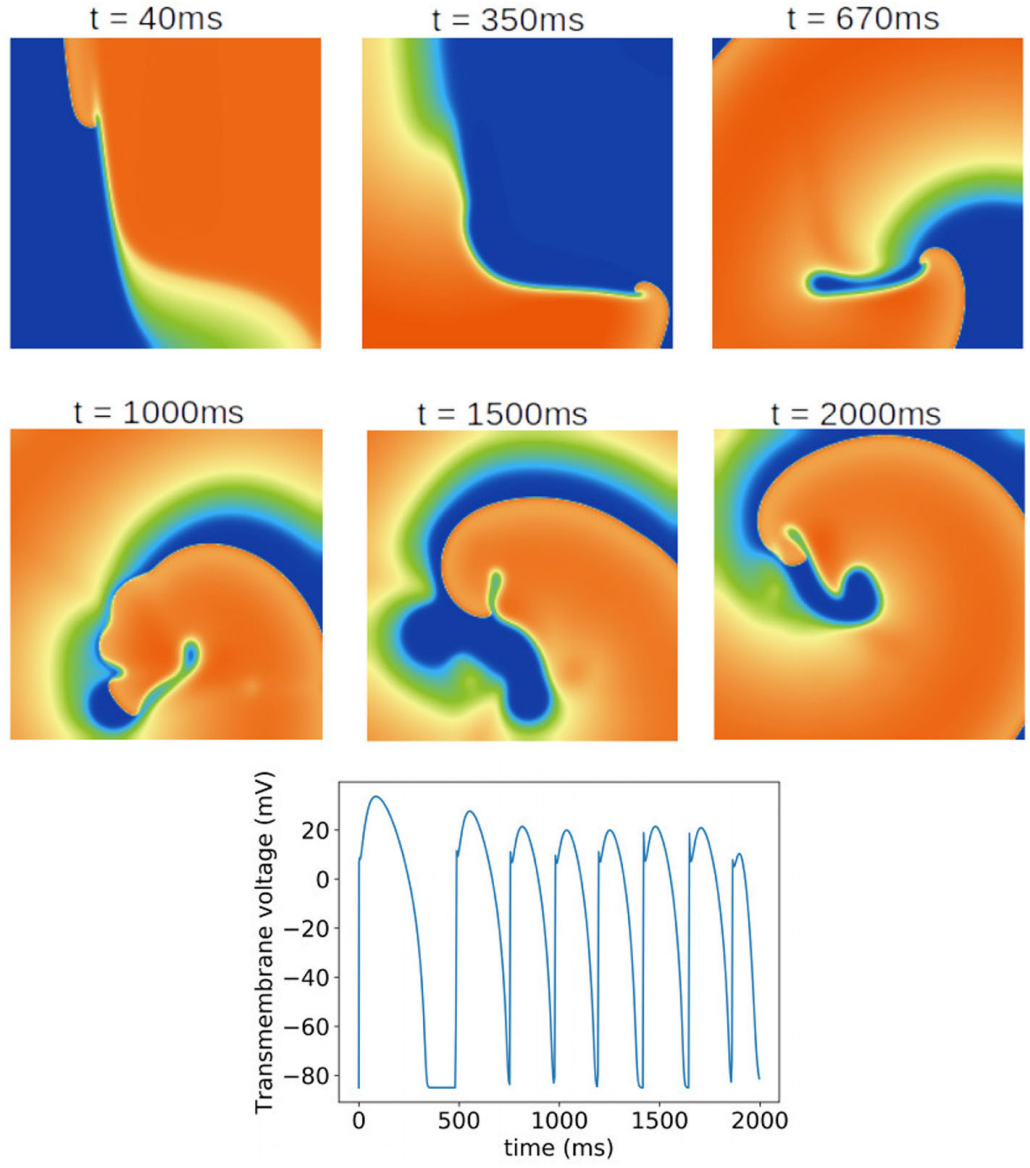
Baseline spiral wave simulation using mean values of all parameters. A rotating spiral wave was induced on a square two-dimensional sheet by setting gradients in voltage and *h* at *t* = 0. **(Top)** Progression of the spiral wave at selected times (color represents transmembrane voltage). Number of phase singularities in snapshots: 40 ms: one; 350 ms: one; 670 ms: one; 1,000 ms: three; 1,500 ms: one; 2,000 ms: three. **(Bottom)** Transmembrane voltage as a function of time for the node at the center of the tissue.

**Figure 8:**
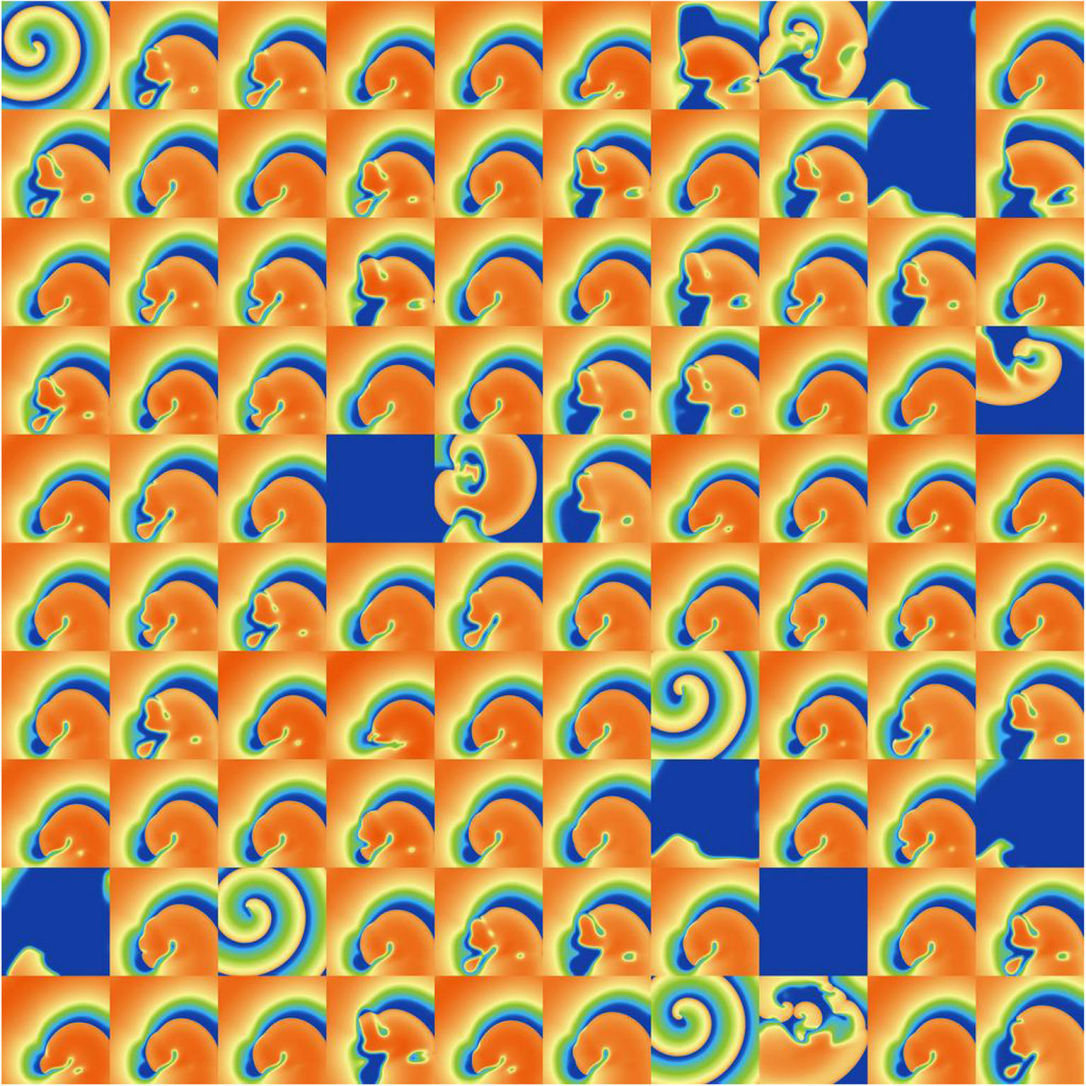
Snapshots of activity at *t* = 1, 000 ms for 100 random parameters, under *I*_to_ parameter uncertainty.

**Figure 9:**
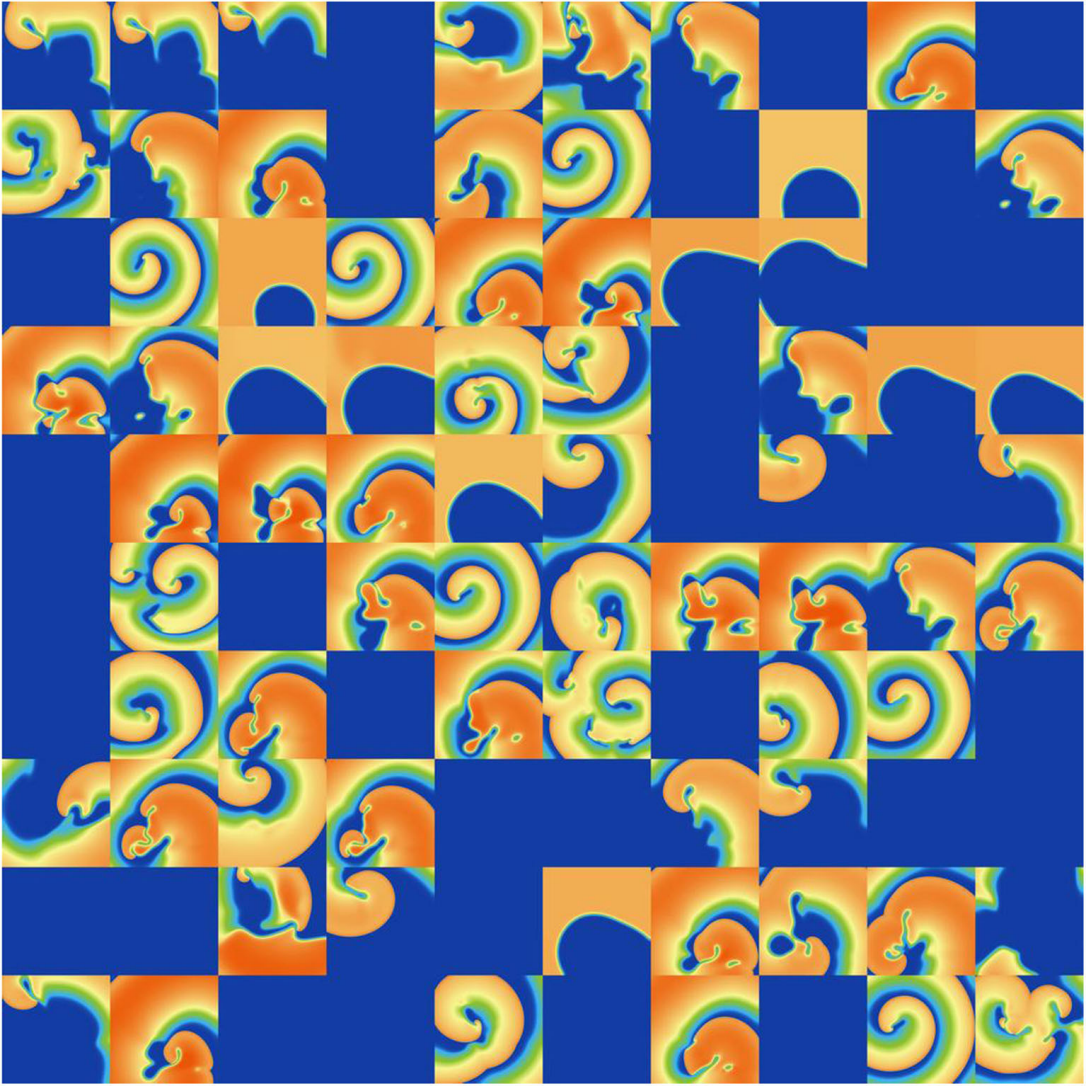
Snapshots of activity at *t* = 1, 000 ms for 100 random parameters, under *I*_CaL_ parameter uncertainty.

**Figure 10:**
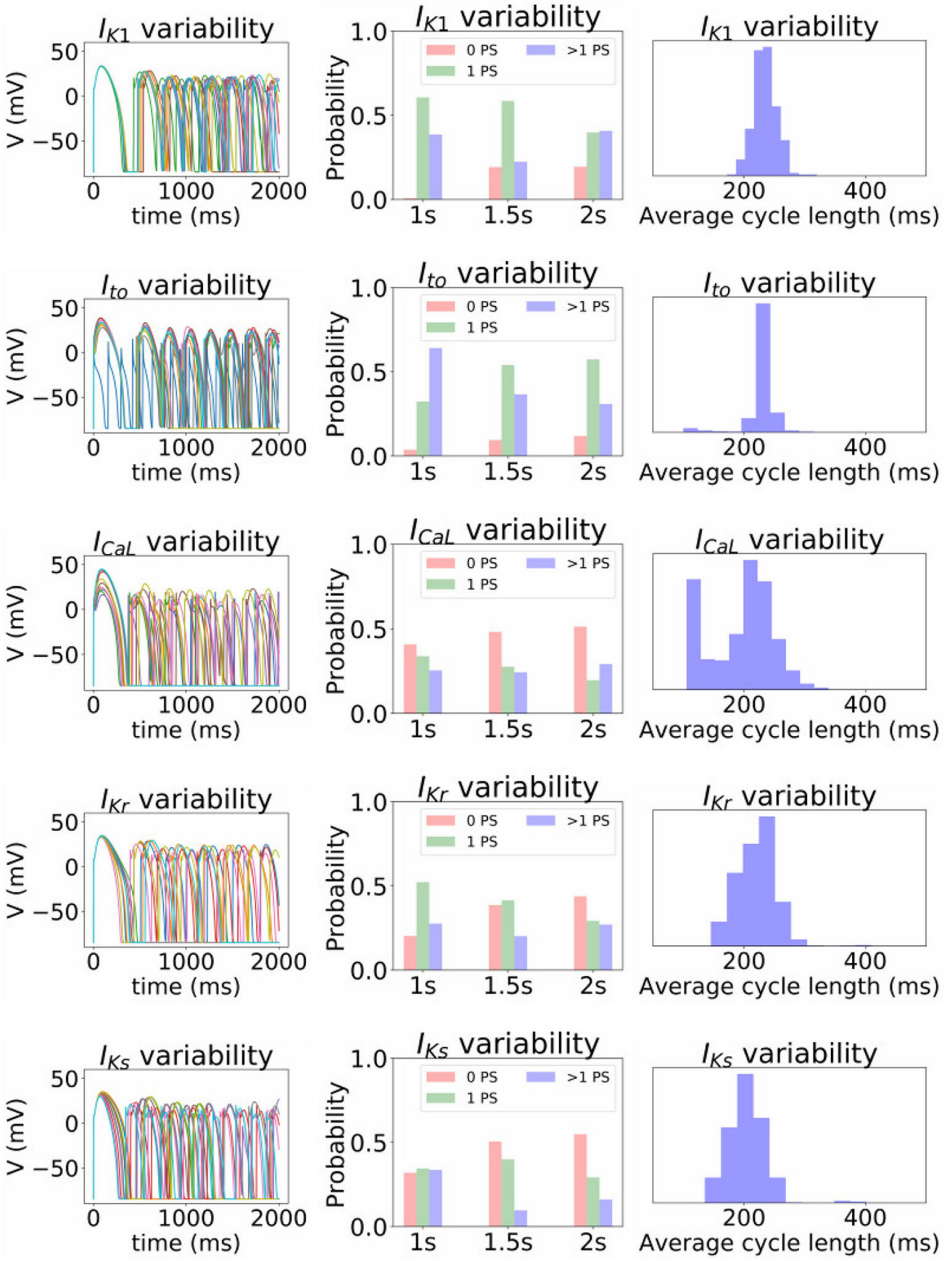
Impact of parameter uncertainty on the spiral wave results. Left column of figures: 10 sample traces of transmembrane voltage for the center node, using random parameters for the current under consideration. Middle column: distribution of number of phase singularities (0, 1, >1) at *t* = 1, 000, 1, 500, and 2, 000 ms (labeled “1s” etc), given the parameter uncertainty. Right column: distribution of average cycle length in the *t* = 1, 000 to *t* = 2, 000 ms window, conditional on at least 1 cycle in this window.

Finally, we investigated the impact of the parameter uncertainty on reentrant spiral wave simulations in tissue (2D, 16 by 16 cm square domain). The results of the baseline simulations are shown in Figure 7, which uses mean values of all parameters. Two seconds of activity were simulated, taking about 40 s of computation using a desktop with a GeForce GTX 960 graphics card with 1,024 CUDA cores (typical utilization: 50–70%). We then considered each current in turn, randomly sampling *N* = 1, 000 parameters for each, with parameters in other currents kept at mean values. Results are presented in Figures 8–10. Figure 8 presents 100 snapshots of the activity at *t* = 1, 000 ms, for 100 of the 1,000 randomly chosen *I*_to_ parameter sets. Figure 9 is the equivalent figure for *I*_CaL_ parameter uncertainty. Corresponding figures for *I*_K1_, *I*_Kr_, and *I*_Ks_, and for all currents at *t* = 2, 000 ms, are provided in the Supplementary Material. Figure 10 presents the distributions of number of phase singularities (PSs) and average cycle length for the node at the center of the domain. This figure also includes 10 random traces for the center node, as a simple visual indication of the output variability.

First, it is interesting that across all the 5,000 simulations, it was possible to induce reentrant activity lasting at least 1,000 ms in about 80% of the simulations, all using the same induction protocol. This observation is based on the number of simulations with one or more phase singularities at *t* = 1, 000 ms, given that zero phase singularities implies no activity (blue subplots in Figures 8, 9) or activity about to die out. This demonstrates that spiral wave inducibility is reasonably robust to the underlying parameter uncertainty, perhaps more so than might have been expected.

Considering first *I*_K1_ and *I*_to_, uncertainty in these parameters had very little impact on inducibility, and limited impact on average cycle length. Figure 8 shows that most of the *I*_to_ simulations had a similar voltage profile after 1,000 ms as the baseline simulation, though there was a small probability of low wavelength circular spirals. We observed in Figure 2C that some random *I*_to_ parameters led to shortened action potentials—it is these parameters which lead to the circular spirals in the 2D simulations. For both *I*_K1_ and *I*_to_ the distributions of average cycle length were similar to the single cell APD distributions (Figures 2B,C), though for *I*_K1_ less skewed and for *I*_to_ more wide-ranging.

*I*_CaL_ uncertainty had the greatest impact on the tissue results, with near 50% of simulations not inducing or dying out by 1,000 ms, and with a bimodal wide-ranging distribution of average cycle length for the remaining simulations. As with *I*_to_, parameters corresponding to shortened action potentials led to circular low-wavelength spirals. Interestingly, highly complex behavior occurred more often under *I*_CaL_ uncertainty than for the other currents: 3% of *I*_CaL_ simulations had >10 PSs at *t* = 2, 000 ms, in contrast to <1% for the other currents (results not plotted). Overall, *I*_CaL_ parameter uncertainty appears to strongly affect the type of reentrant behavior; the results again emphasize the importance of this current on model results and the need to reduce or at least better characterize uncertainty in its parameters. Finally, *I*_Kr_ and *I*_Ks_ had a moderate impact on the tissue simulations: uncertainty in these parameters had less of an effect than *I*_CaL_ parameters but more of an effect than *I*_K1_ and *I*_to_ parameters, for example activity had died out after 2 s in about half *I*_Kr_ and *I*_Ks_ simulations.

## 4. Discussion

### 4.1. Summary of Results

In general data-driven, comprehensive (in the sense of considering all parameters) UQ in cardiac modeling has been considered infeasible, due to the complexity of cardiac cellular models. All previous work in this domain has either limited the uncertain parameters to a subset (usually minority subset) of the total number of parameters, and/or (usually and) prescribed the parameter uncertainty rather than estimating it from experimental data. The aim of this work was to quantify the uncertainty, due to population variability, in repolarization parameters of a canine cardiac action potential model, and to understand the subsequent uncertainty in model predictions. Model outputs that were studied included the action potential, relative effects on action potential duration of drug-induced current block, and “biomarkers” of reentrant behavior in tissue simulations. Our results demonstrate for the first time the feasibility of comprehensive data-driven cardiac UQ (though we only accounted for uncertainty in repolarization parameters; *I*_Na_ parameters were left fixed).

A variety of approaches were used to characterize uncertainty in the repolarization model parameters: (i) fitting parameters to data derived from a number of individual cells; (ii) back-calculating standard deviations from reported parameter standard errors; (iii) fitting parameters with uncertainty, to observable data (e.g., peak current) given or after back-calculating uncertainty in those observables; and (iv) specifying a conservative estimate of uncertainty based on uncertainty in related parameters for other currents. Interestingly, the different methods (i)–(iii) provided similar estimates of uncertainty for related parameters (i.e., steady-state activation/inactivation voltages; time constants; conductances). The same is true of method (iv) by definition. The ensuing parameter uncertainty was substantial, and greater than that typically prescribed when arbitrary variation is used [including our previous work (Pathmanathan et al., 2019)]. When the parameter uncertainty was propagated through the AP model, there was substantial uncertainty in predicted action potentials, which was not surprising given our previous results in Pathmanathan et al. (2019) where even moderate prescribed parameter uncertainty led to significant output uncertainty. *I*_K1_ parameter uncertainty had least impact on the AP; followed by *I*_Kr_. For both these currents, AP shape was relatively robust (invariant) to the underlying uncertainty and no anomalous APs arose. *I*_Ks_ parameter uncertainty led to surprisingly large output uncertainty, although this was caused by large uncertainty in one parameter that was most likely dominated by experimental measurement error rather than genuine population variability (see section 3.1). When considering uncertainty in *I*_to_ parameters, there was a large probability of little impact on AP shape, and a small probability of loss of spike and dome morphology and shortened APs. Uncertainty in *I*_CaL_ parameters strongly affected APs, including loss of spike and dome morphology and shortened APs, early-after-depolarizations and repolarization failure (Figure 2D). These results suggest that when experimental resources are limited, focused experiments to better characterize *I*_CaL_ parameter variability may be the best use of resources.

We expected that *relative* differences, such as change in APD under drug-induced current block, would be more robust to the underlying uncertainty, even if absolute values of model outputs, such as APD, exhibited large uncertainty. Surprisingly, this was not the case; the former exhibited similar magnitudes of uncertainty as the latter. While this is an unfortunate result, analyses such as that performed in Figure 6 could be used to identify level of block at which response is relatively robust to underlying uncertainty. For example, 50% appears a reasonable choice based on Figure 6. Additionally, Figure 6 reveals the currents (*I*_CaL_ and *I*_to_) that it may be important to focus experimental investigation when attempting to minimize output uncertainty.

We had limited data available for building parameter correlation into our statistical model; covariance was included for *I*_K1_ and some *I*_to_ parameters only. However, we investigated the importance of correlation, by removing correlation from *I*_K1_ or *I*_to_, and by artificially introducing it to two *I*_CaL_ parameters. We observed a clear impact of the correlation: *I*_K1_ correlation was responsible for the model exhibiting realistic AP shape, and the introduction of hypothesized *I*_CaL_ correlation reduced the probability of anomalous APs occurring. This strongly supports the expectation that parameter correlation exists in reality. Moreover, the results suggest correlation will often need to be accounted for in cardiac electrophysiological modeling if population variability is modeled. Note that we only considered intra-current parameter correlation, but there is also evidence of inter-current parameter correlation, especially in maximal conductances due to co-expression of currents. For example, Milstein et al. (2012) shows potential correlation between *g*_Na_ and *g*_K1_ in rat, Rees et al. (2018) presents evidence for correlation between *g*_CaL_ and potassium current conductances in mouse, and recently Ballouz et al. (2019) analyzed RNA-Seq datasets to identify correlation between *g*_CaL_ and *g*_Kr_ in human. Fully characterizing such relationships will likely be one of the biggest challenges in cardiac AP model UQ.

Note that our ability to draw *direct* conclusions about human AP models based on these results is limited. While canine is frequently used as an experimental model for human, there are known differences which limit the ability to extrapolate even average behaviors from canine to human, for example related to *I*_to_ characteristics (Akar et al., 2004) or smaller *I*_K1_, *I*_Kr_, and *I*_Ks_ in human vs. dog which results in a lower repolarization reserve (Jost et al., 2013). Species differences in regards to variability are even more uncertain, and it is difficult to estimate the extent to which the conclusions in this work (importance of *I*_CaL_ variability on action potential, potential importance of parameter correlation for physiological behavior, and uncertainty in relative model outputs) will apply to human action potential models. Further research is needed to verify if the findings apply to human models, but the methods and workflow presented in this paper provide a pathway to begin such research. Our results do suggest that appealing to the fact that a quantity of interest is only a relative difference is not a particularly strong justification for not performing UQ (including for human models), in the absence of other evidence.

### 4.2. Comparison With Other Work

It is worth comparing our approach with related cardiac UQ efforts. In this sub-section we focus on the method used to to characterize parameter variability; see Pathmanathan et al. (2019) for a comparison with other publications in regards to the number of parameters varied. As stated earlier, it is common to introduce arbitrarily-chosen uncertainty in parameters when studying impact of parameter uncertainty on cardiac AP model predictions (e.g., Sadrieh et al., 2014; Chang et al., 2015; Hu et al., 2018; Ballouz et al., 2019). We are not aware of other analyses that propagate entirely data-driven (not arbitrarily-chosen) independently-derived parameter uncertainty though a cardiac AP model, apart from our initial work on this subject for *I*_Na_ inactivation (Pathmanathan et al., 2015), and articles that consider uncertainty in non-endogenous parameters [e.g., drug-response parameters (Chang et al., 2017; Costabal et al., 2019)]. There are also methods that use the full action potential model for parameter uncertainty estimation, such as traditional Bayesian calibration methods (Houston et al., 2020), history-matching approaches (Coveney and Clayton, 2018), and the recent population of models (POM) approach (Britton et al., 2013; Gong et al., 2020), all of which are related (Whittaker et al., 2020). Bayesian calibration methods compute a posterior distribution for parameter values. The interpretation of this distribution depends on the type of data the model is fit to. If the model is fit to single values of an output quantity (e.g., APD90), the posterior distribution represents calibration uncertainty (similar to the ellipses in Figure 1C) (see e.g., Houston et al., 2020). If the model is fit to a distribution of the output quantity, based on information on how the quantity varies across a population, the posterior distribution represents parameter variability (and calibration uncertainty). The POM approach is similar, and involves specifying bounds on quantities of interest that are considered physiologically reasonable, and retaining points in parameter space, from a pre-selected region, for which the model output falls within the pre-specified range. This has recently been extended to specifying output distributions instead of just bounds, and computing a multivariate parameter distribution (Lawson et al., 2018), which could be considered analogous to the parameter distributions we derived (Appendix). The key difference between the POM approach and our approach used here is that we aimed to avoid using the action potential model to quantify parameter uncertainty—all parameter uncertainty estimates were based on independent data or comparison to related parameters (section 2.2.4). This was because our focus was *model evaluation*, rather than *model application*; specifically we wanted to understand the impact of parameter uncertainty on the model, which would not be possible had we used to the model to compute parameter uncertainty. The POM approach does not test the model, and in fact the reliability of the parameter distributions obtained is founded upon the reliability of the model. Our approach is especially a “bottom-up” forward UQ analysis, whereas the Bayesian calibration/POM approach is a backward UQ which has the potential for inferring true parameter distributions (especially for parameters which are difficult to measure experimentally) and correlations between parameters (especially inter-current correlations), but is heavily reliant on confidence in the model equations. Ultimately, both approaches have value in different stages of the model development and application workflow. One possibility is using the bottom-up forward UQ approach in iterative model development and experimental resource allocation, with the resultant probability distributions used to inform the region of parameter space to be considered in a subsequent backward UQ stage.

### 4.3. Limitations

There are various limitations to the methods applied in this paper. First, we used relatively crude methods to estimate population variability for several parameters, such as back-calculating standard deviation from reported standard errors, and we assumed the distribution shape (normal or log-normal) rather than inferring from the data, or treating the uncertainty as epistemic and using interval analysis (Oberkampf et al., 2004). We consider these reasonable choices given the scarcity of relevant data, because this paper is the first time such an analysis has been attempted and there were dozens of parameters that needed to be considered. It is plausible however that many of distributions we provided in the Appendix are wider-ranging than in reality, and there is considerable potential for refinement with focused experimentation and more sophisticated analysis. Moreover, we neglected the impact of experimental bias and measurement error. Experimental variability may be responsible for some or much of the observed variability (Lei et al., 2020a). A second limitation is the simple two-step method used to estimate the population mean and covariance matrices, for currents (*I*_K1_ and *I*_to_) where cell-wise data was available. This approach does not account for calibration uncertainty (ellipses in Figure 1C. A better approach is to use a hierarchical method, such as non-linear mixed effects which would essentially down-weight cells for which there is substantial calibration uncertainty (large ellipses) when inferring the population mean and variance. Another limitation is the fact that we considered each current in isolation when propagating the uncertainty through the model. This is at odds with the global sensitivity analysis paradigm in which the full parameter space given the specified probability distributions should be explored. We could have performed a global sensitivity analysis of the results of Figure 2A (where all parameters were varied), and then assessed the contributions of each of the different currents to the range of APs observed (non-behavioral analysis would also have been needed, since repolarization failure occurred; see Pathmanathan et al., 2019). However, we believe it is more intuitive and useful in an initial analysis (and therefore appropriate for this paper) to consider each of the currents in isolation. Finally, it should be noted that the simplicity of the model impacts the type of variability observed, for example there was little possibility of significant variability in resting membrane potential because the model did not include any exchanger or background currents and because we did not allow environmental parameters (*E*_K_, *E*_Na_, *E*_Ca_) to vary.

### 4.4. Outlook

The ultimate goal of this line of research is the development of AP models with parameter uncertainty quantified in the form of probability distributions, rather than parameters taking fixed values, ideally together with the model and parameter distributions being such that the resultant output uncertainty matches that observed in reality–a very strong level of validation. Achieving this admittedly ambitious goal will require refinement of simplified models, such as that used here, and more accurate characterizations of parameter uncertainty (see limitations above; our initial estimates of parameter uncertainty are quite crude). It is also likely that “model discrepancy”–the difference between model equations and reality due from limitations in (or incorrect) model form–will need to be characterized using formal model discrepancy techniques (Lei et al., 2020b), since simplified models for which comprehensive UQ is feasible generally exhibit greater discrepancy (Huberts et al., 2018). It is worth noting that these approaches are relevant to patient-specific models too. With rare exceptions, patient-specific models contain parameters that are not personalized to patient data, in addition to the personalized parameters (Gray and Pathmanathan, 2018). The non-personalized parameters are usually fixed to population average values, but in principle it is better, and more aligned to the UQ paradigm, to characterize them using probability distributions that account for population variability (ideally, probability distributions conditional upon the available patient data, such as sex or age or covariates that are personalized). Statistical representations of population variability will be useful even for parameters that will be personalized to patients, because those distributions can serve as informative priors in Bayesian calibration.

### 4.5. Conclusion

Overall, our results provide for the first time information on the expected uncertainty in cardiac AP and spiral wave models, given experimentally-derived parameter uncertainty in all repolarization parameters. We have demonstrated that robustness of model outputs to parameter uncertainty should not be assumed, even for model outputs that are relative differences. Our results begin to reveal the role of correlation between parameters in the cardiac action potential. We have demonstrated that it is feasible to perform data-driven forward uncertainty quantification in cell model parameters considering uncertainty in the vast majority of parameters, including assessment of the impact of uncertainty in computationally-demanding tissue simulations. We believe that use of the methods and approaches presented here will lead to improved models and ultimately more reliable decision-making, when cardiac models are used in clinical, regulatory, or product development applications.

## Data Availability Statement

All data used in model generation is already published (see text), except that used for parameterizing the *I*_K1_ model (see Table 1). This data is available upon result.

## Disclosure

The mention of commercial products, their sources, or their use in connection with the material reported herein is not to be construed as either an actual or implied endorsement of such products by the Department of Health and Human Services.

## Author Contributions

PP devised the project, implemented the simulation and analysis code, ran the simulations and analysis, and wrote the paper. SG implemented and ran the analysis code, and reviewed the paper. JC provided the experimental data and reviewed the paper. AK and FF provided the simulation code and reviewed the paper. RG developed the model and provided the feedback on all aspects of the project. All authors contributed to the article and approved the submitted version.

## Conflict of Interest

The authors declare that the research was conducted in the absence of any commercial or financial relationships that could be construed as a potential conflict of interest.

## Footnotes

^1^https://clinicaltrials.gov/ct2/show/NCT03536052

## Appendix

In the below conductances *g*_*X*_ have units mS/cm^2^, half (in)activation voltages *E*_*X*_ have units mV, slopes *k*_*X*_ have units mV, and time constants $\tau_{X}^{*}$ have units ms.

For *I*_K1_:

(A1) $$\begin{array}{l} \left[\begin{array}{c} \log\left(g_{\text{K}1}\right)\\ E_{z}\\ \log\left(k_{z}\right) \end{array}\right]\sim N\left(\mu ,\Sigma \right)\text{ with }\mu =\left[\begin{array}{c} -0.296\\ -92.1\\ 2.51 \end{array}\right],\\ \;\Sigma =\left[\begin{array}{ccc} 0.0758 & -0.694 & -0.00653\\ -0.694 & 25.1 & -0.362\\ -0.00653 & -0.362 & 0.0192 \end{array}\right] \end{array}$$

For *I*_to_:

$$\begin{array}{l} \left[\begin{array}{c} \log\left(g_{\text{to}}\right)\\ E_{r}\\ \log\left(k_{r}\right) \end{array}\right]\sim N\left(\mu ,\Sigma \right)\text{ with }\mu =\left[\begin{array}{c} -1.83\\ 14.4\\ 2.46 \end{array}\right],\\ \;\Sigma =\left[\begin{array}{ccc} 0.143 & 0.377 & -0.0257\\ 0.377 & 33.4 & 0.493\\ -0.0257 & 0.493 & 0.0191 \end{array}\right]\\ \left[\begin{array}{c} E_{s}\\ \log\left(k_{s}\right) \end{array}\right]\sim N\left(\mu ,\Sigma \right)\text{ with }\mu =\left[\begin{array}{c} -48.1\\ 1.16 \end{array}\right],\\ \;\Sigma =\left[\begin{array}{cc} 37.4 & 0.262\\ 0.262 & 0.0515 \end{array}\right]\\ \;\tau_{s}^{*}\sim \text{lognormal}\left(\mu ,\sigma^{2}\right)\;\mu =2.29,\sigma =0.129 \end{array}$$

For *I*_CaL_:

$$\begin{array}{l} g_{\text{CaL}}\sim \text{lognormal}\left(-2.29,0.341^{2}\right)\\ \;E_{d}\sim N\left(0.7,3.71^{2}\right)\\ \;k_{d}\sim \text{lognormal}\left(1.44,0.171^{2}\right)\\ \;E_{f}\sim N\left(-15.7,7.01^{2}\right)\\ \;k_{f}\sim \text{lognormal}\left(1.52,0.134^{2}\right)\\ \;\tau_{f}^{*}\sim \text{lognormal}\left(3.37,0.303^{2}\right) \end{array}$$

For *I*_Kr_:

$$\begin{array}{l} E_{xr}\sim N\left(-26.6,4.43^{2}\right)\\ k_{xr}\sim \text{lognormal}\left(1.86,0.145^{2}\right)\\ \;E_{y}\sim N\left(-49.6,7.80^{2}\right)\\ \;k_{y}\sim \text{lognormal}\left(3.15,0.0638^{2}\right) \end{array}$$

For *I*_Ks_:

$$\begin{array}{l} E_{xs}\sim N\left(24.6,33.9^{2}\right)\\ k_{xs}\sim \text{lognormal}\left(2.48,0.140^{2}\right)\\ \tau_{xs}^{*}\sim \text{lognormal}\left(6.42,0.220^{2}\right) \end{array}$$
