# Supplementary material for: Data-Driven Uncertainty Quantification for Cardiac Electrophysiological Models: Impact of Physiological Variability on Action Potential and Spiral Wave Dynamics
Source: Front Physiol. 2020 Nov 19;11:585400. doi: 10.3389/fphys.2020.585400 (PMC7711195; doi:10.3389/fphys.2020.585400)
Supplement: Supplementary file 1 [file Data_Sheet_1.pdf]

Supplementary Material: Data-driven uncertainty quantification for cardiac electrophysiological models: impact of physiological variability on action potential and spiral wave dynamics

1 RESULTS FROM STAGE 1 OF THE TWO-STAGE APPROACH

Figure S1 presents results of fitting

$$F(g_{\text{to}}, E_r, k_r) = g_{\text{to}} \left(1 + \exp\left(\frac{-(V - E_r)}{k_r}\right)\right)^{-1} (V - E_{\text{K}})$$

to voltage clamp recordings from $n = 16$ canine epicardial cells. E_{K} was set to -85 mV.

Figure S2 presents results of fitting

$$F(E_s, k_s, \alpha) = \left(\frac{1}{1 + \exp((V - E_s)/k_s)} + \alpha\right)(1 + \alpha)^{-1};$$

to voltage clamp recordings from $n = 14$ canine epicardial cells.

2 BIMODAL APD HISTOGRAM UNDER UNCERTAIN I_{KS} PARAMETERS

Figure S3 plots the relationship between E_{xs} and APD for the uncertain I_{Ks} parameters experiment (see main body, Figure 2(F)). The figure shows that the value of E_{xs} is a dominant factor in determining APD, as compared to the other I_{Ks} parameters (compare red diamonds with blue stars), and that at larger values of E_{xs} the relationship between E_{xs} and APD is flat.

3 IMPACT OF PARAMETER CORRELATION ON I_{CAL} WINDOW CURRENT

Figure S4 plots the impact of introducing correlation between I_{CaL} parameters E_d and E_f, on the magnitude and location of the I_{CaL} window current, to complement Figure 3 in the main body. Given a set of I_{CaL} parameters $(g_{\text{CaL}}, E_d, k_d, E_f, k_f)$, window current magnitude was defined as the maximum absolute value of $g_{\text{CaL}} d_\infty(V; E_d, k_d) f_\infty(V; E_f, k_f)(V - E_{\text{Ca}})$; location (center) was defined as the voltage at which the maximum was attained. The figure plots the distribution of the magnitude and location under uncertain parameters, for different levels of correlation between E_d and E_f.

4 IMPACT OF I_{TO} PARAMETER CORRELATION

Figure S5 plots the results of removing I_{to} parameter correlation on action potentials and APD distribution, that is, repeating the simulations behind Figure 2(C) with a diagonal covariance matrix for I_{to} parameters.

5 SNAPSHOTS OF ACTIVITY IN SPIRAL WAVE SIMULATIONS UNDER PARAMETER VARIABILITY

For the spiral wave uncertainty analysis, Figures S6-S8 presents 100 snapshots of the activity at $t = 1000$ms, for 100 randomly chosen parameters, under I_{K1}, I_{Kr} and I_{Ks} uncertainty, respectively. Corresponding figures for I_{to} and I_{CaL} are in the main body. Figures S9-S13 are the corresponding figures for $t = 2000$ms, for all five currents.

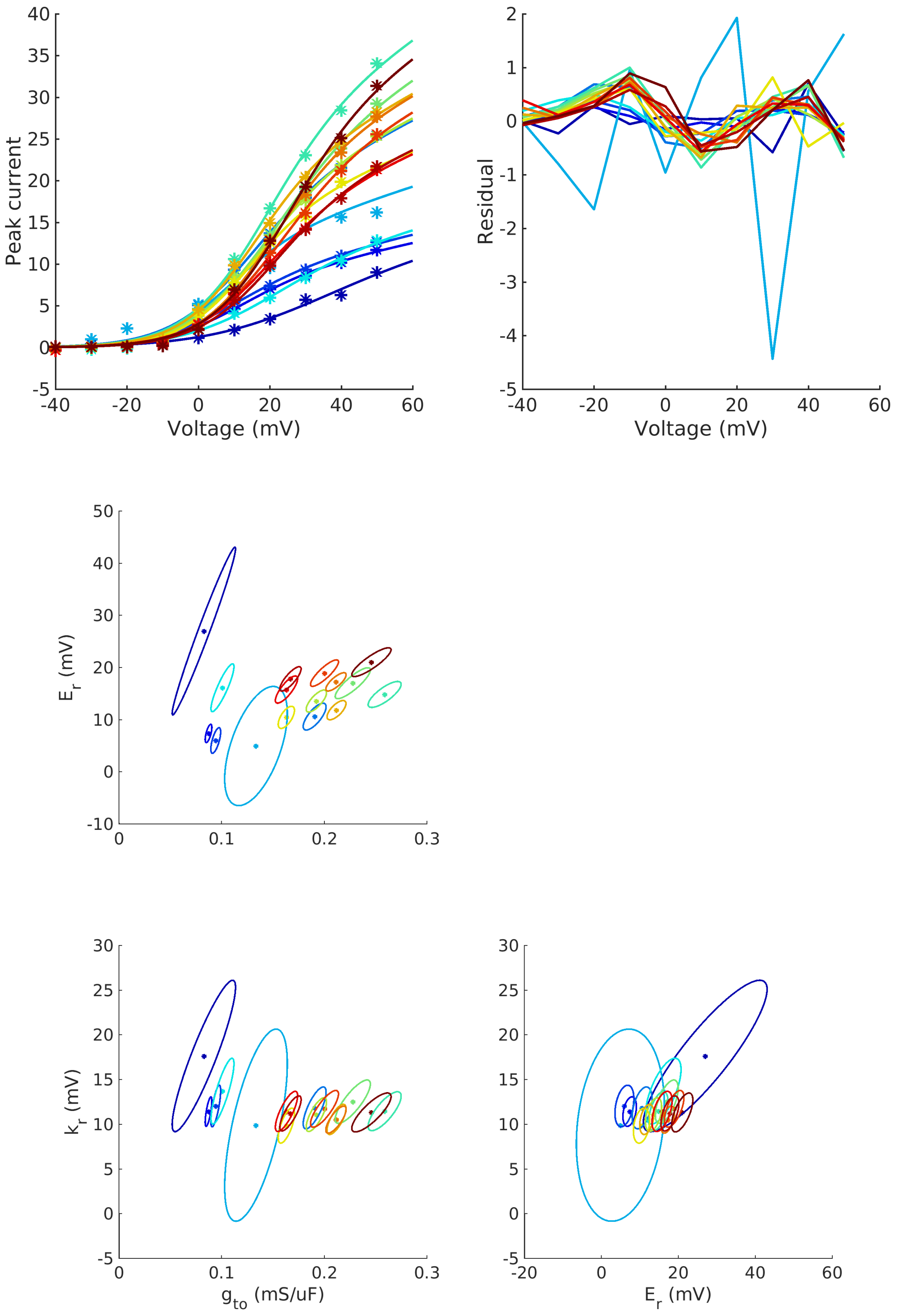

Figure S1. Individualized fits of I_{to} activation parameters. Top-left: experiment voltage clamp data (stars) and fitted model (line) for $n = 16$ cells. Top-right: corresponding residuals. Bottom-half: cell-specific parameters (solid diamonds) and cell-specific 90% confidence regions (ellipses) for the three fitted parameters, g_{to}, E_r, k_r, for each of the cells.

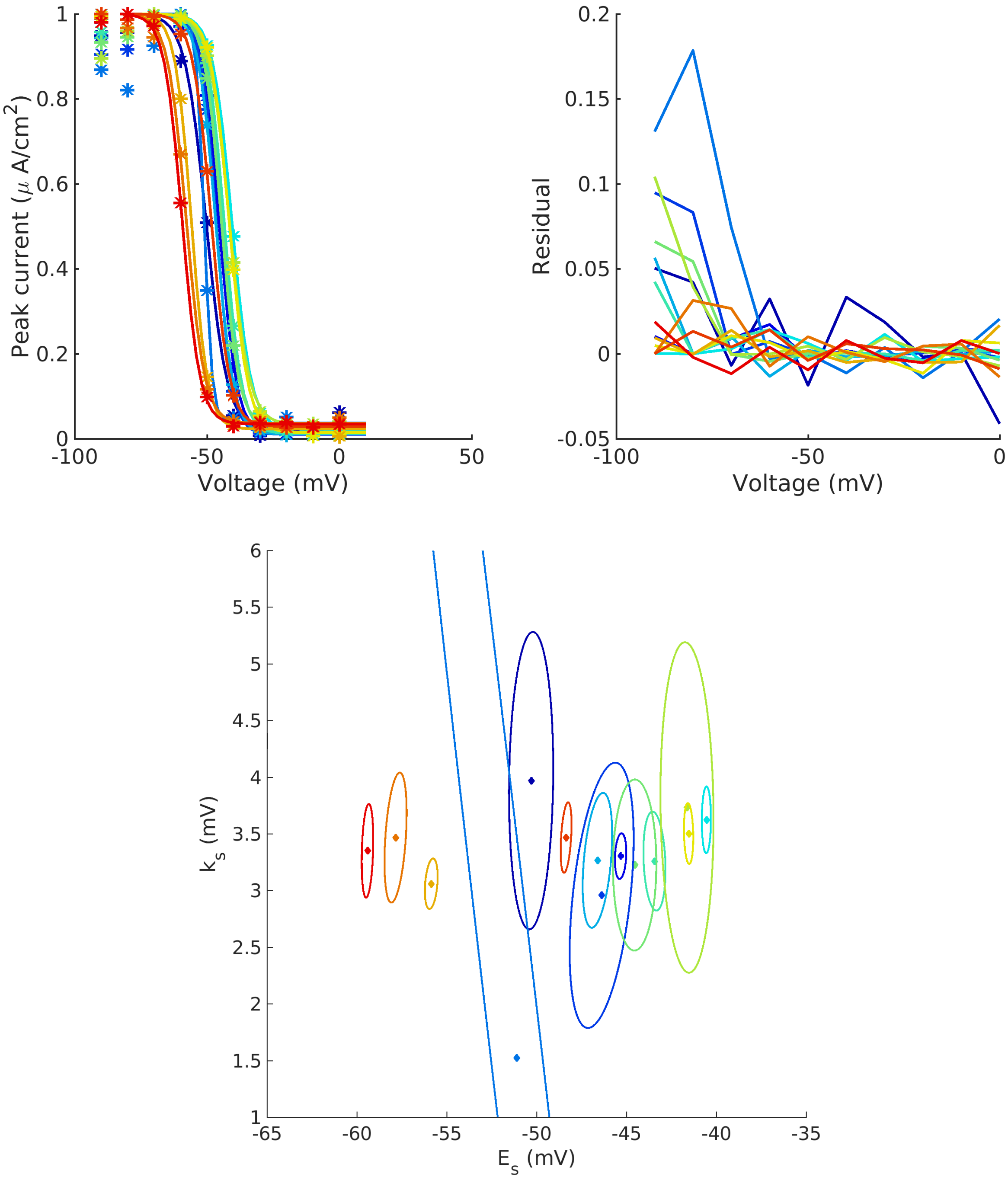

Figure S2. Individualized fits of I_{to} inactivation parameters. Top-left: experiment voltage clamp data (stars) and fitted model (line) for $n = 14$ cells. Top-right: corresponding residuals. Bottom: cell-specific parameters (solid diamonds) and cell-specific 90% confidence regions (ellipses) for two (E_s, k_s) of the three fitted parameters (offset α was fit and then discarded).

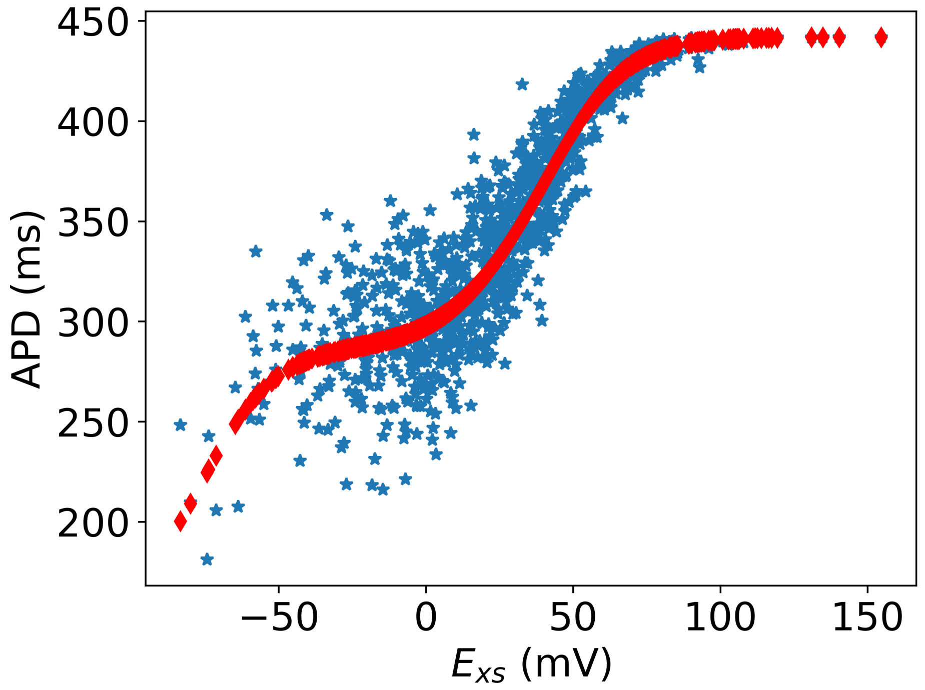

Figure S3. Relationship between E_{xs} and APD. Blue stars represent simulations where other I_{Ks} parameters were uncertain. Red diamonds represent simulations where other I_{Ks} parameters are fixed at mean values.

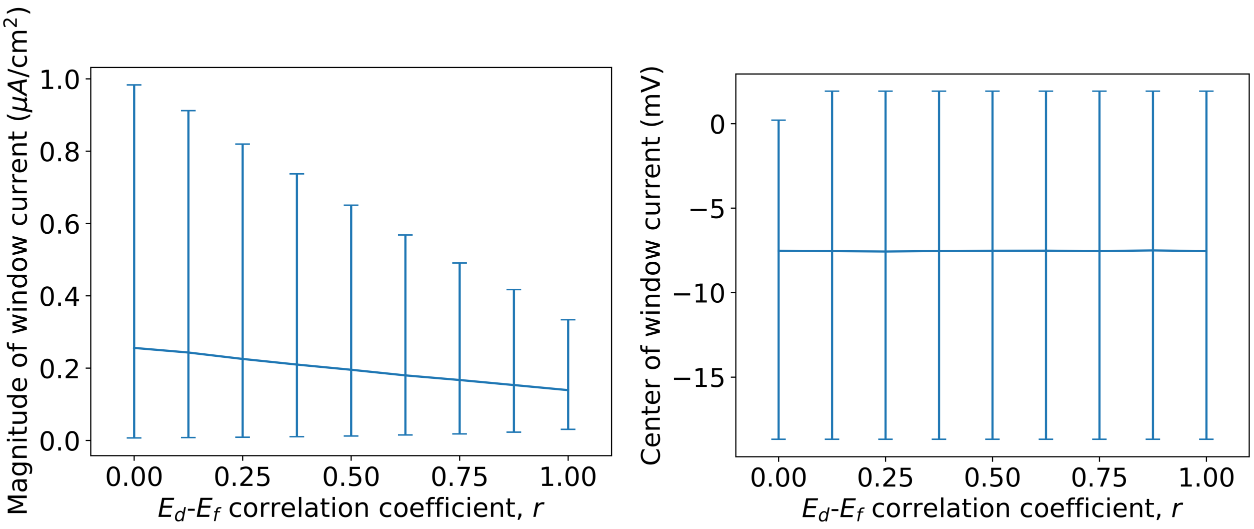

Figure S4. Effect of introducing correlation between half-activation voltage E_d and half-inactivation voltage E_f on I_{CaL} window current. Left: mean value of window current magnitude with 5th and 95th percentiles. Right: mean value of window current location with 5th and 95th percentiles.

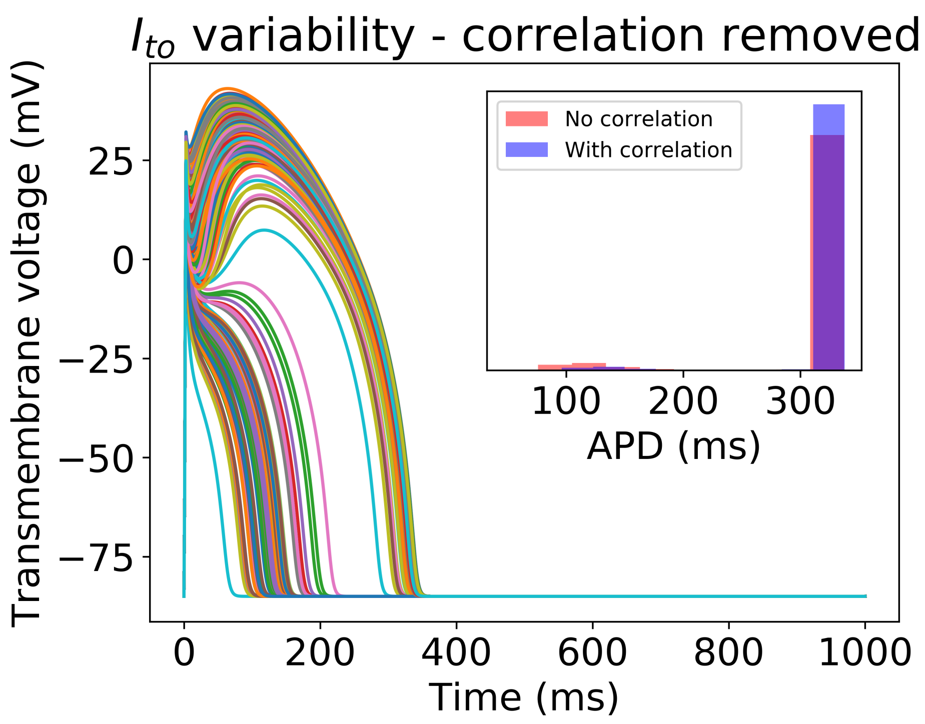

Figure S5. Effect of neglecting inferred correlations in I_{to} parameters. 1000 sample action potentials with histograms of APD ($N = 100,000$) in inset.

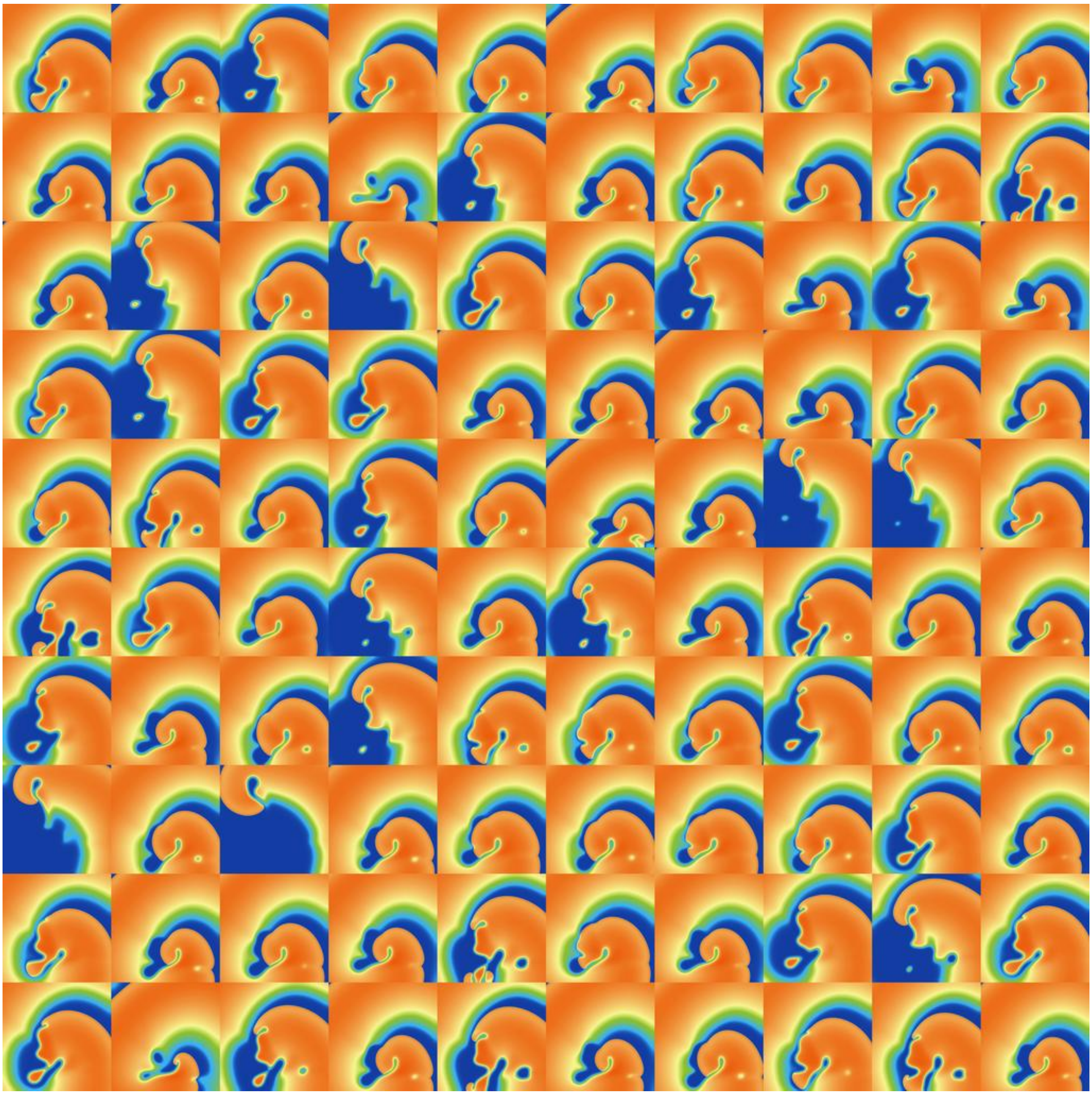

Figure S6. Snapshots of activity at $t = 1000$ms for 100 random parameters, under I_{K1} parameter uncertainty.

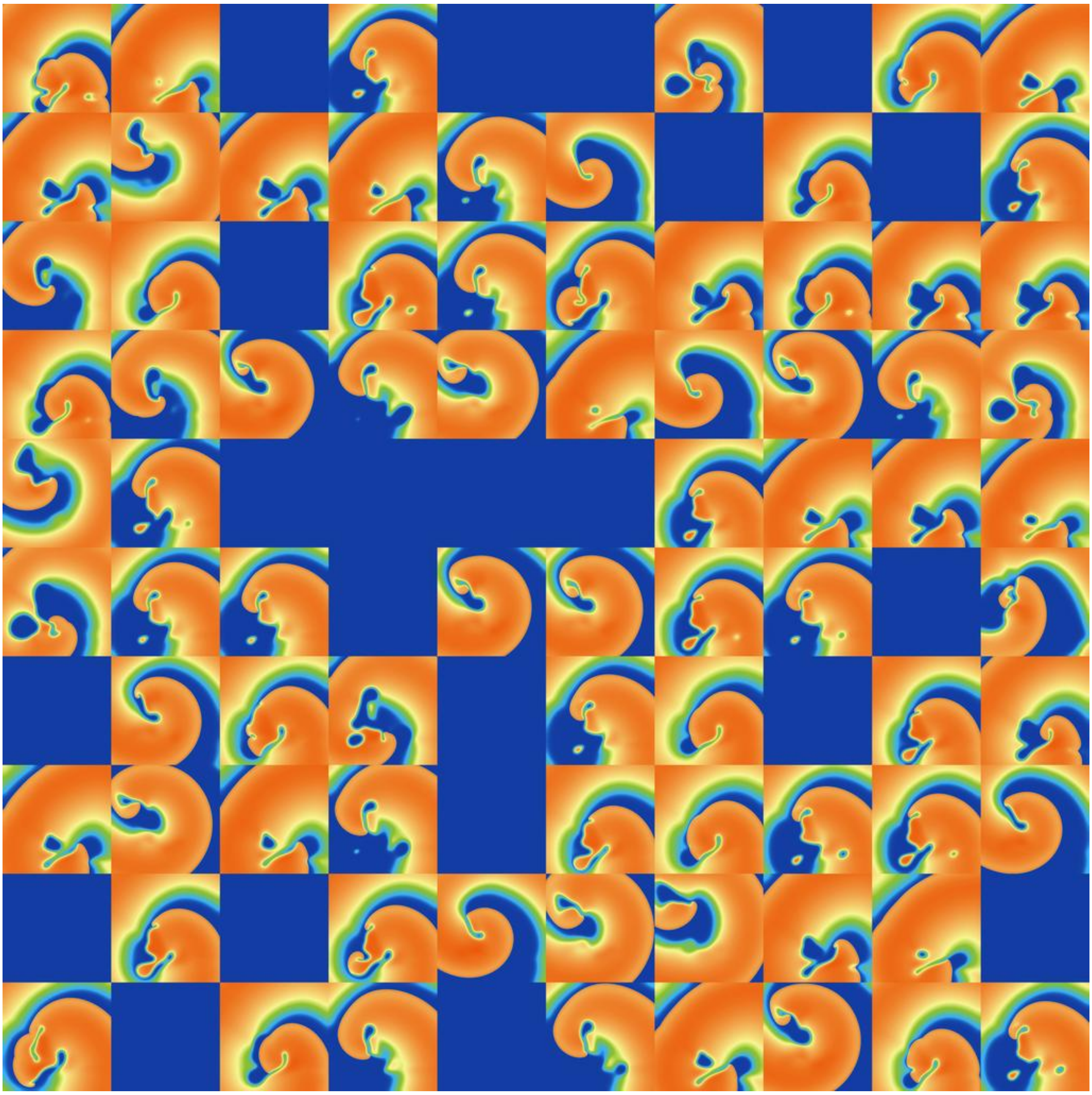

Figure S7. Snapshots of activity at $t = 1000$ms for 100 random parameters, under I_{Kr} parameter uncertainty.

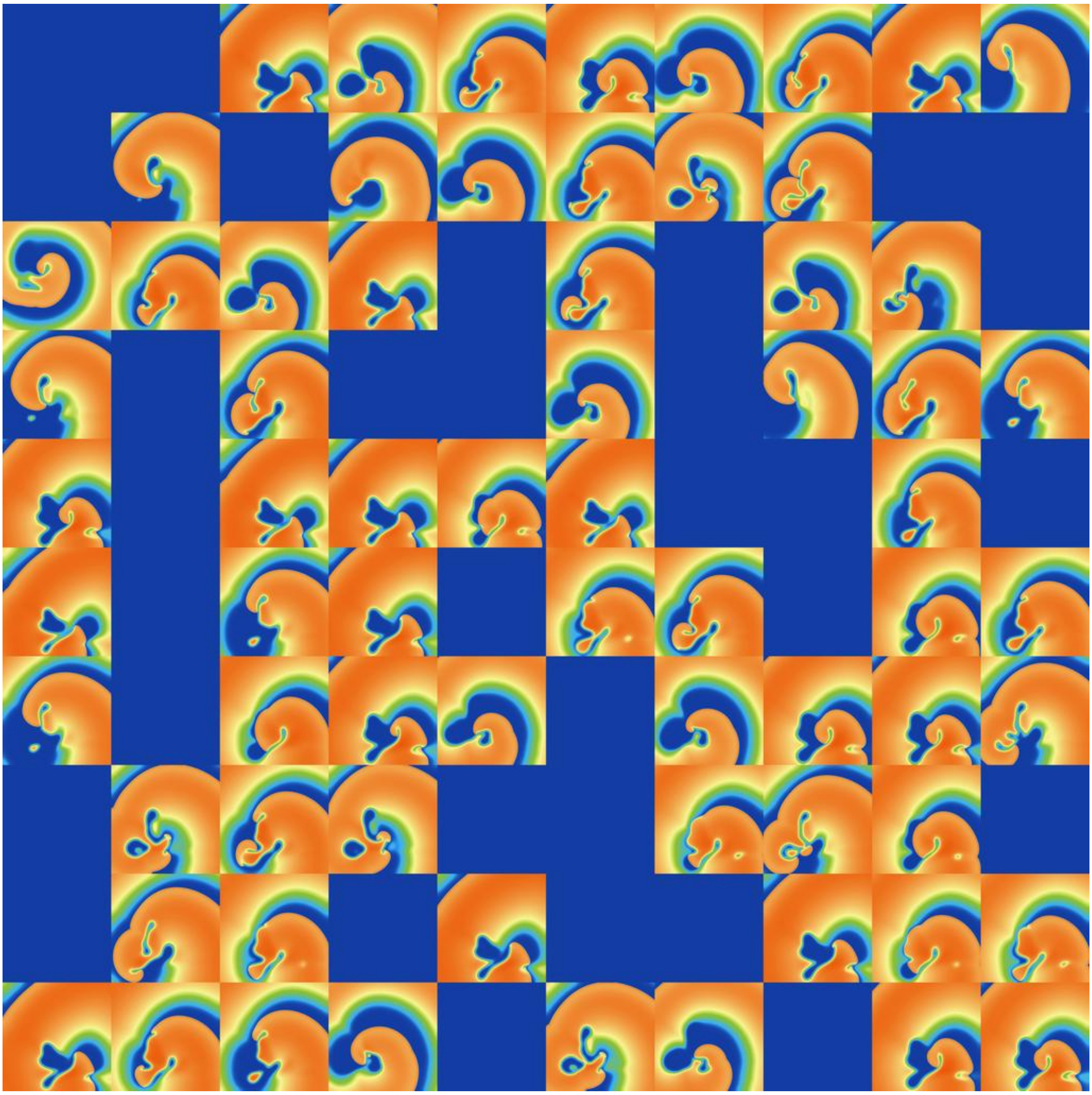

Figure S8. Snapshots of activity at $t = 1000\mathrm{ms}$ for 100 random parameters, under I_{Ks} parameter uncertainty.

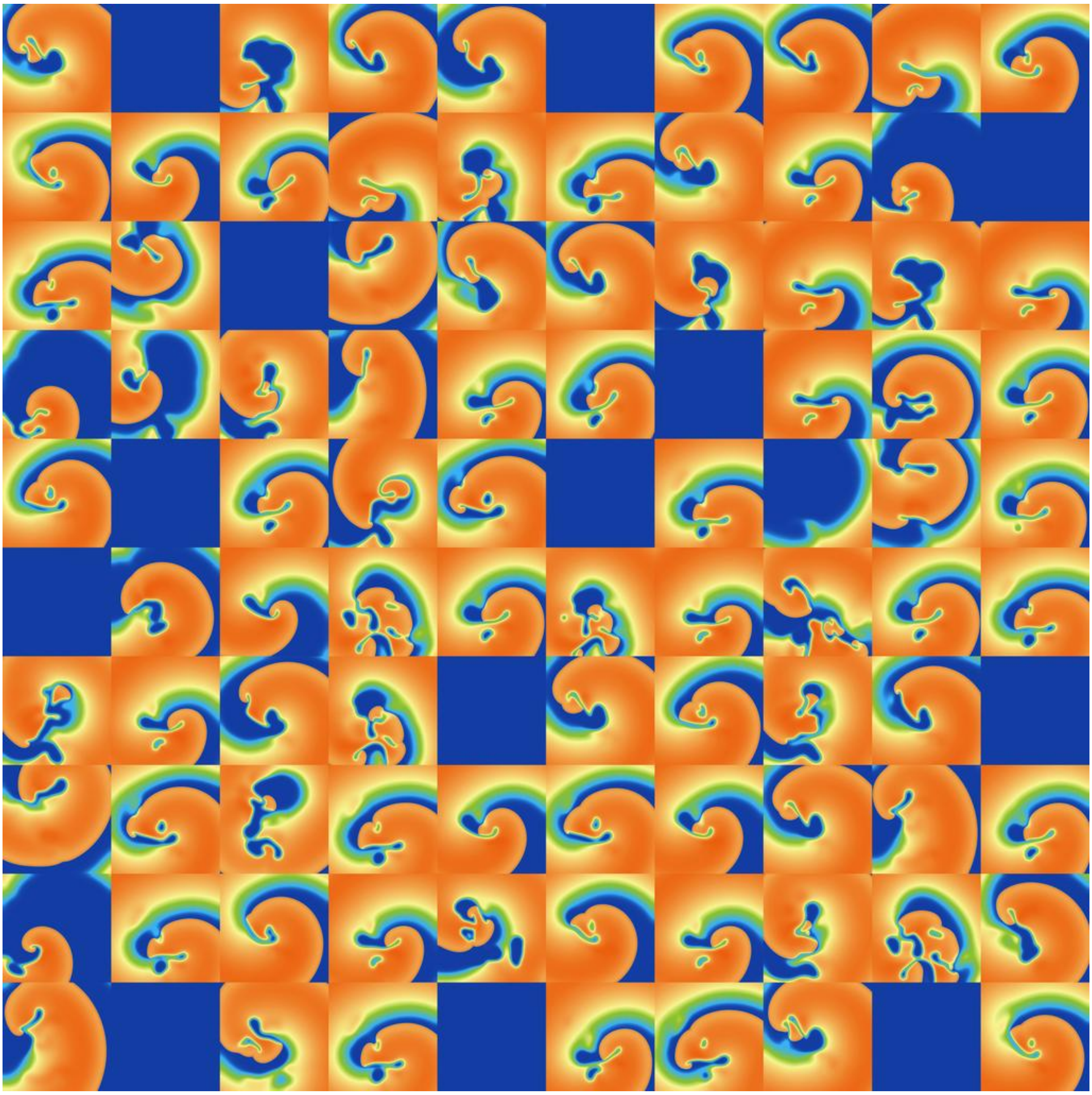

Figure S9. Snapshots of activity at $t = 2000$ms for 100 random parameters, under I_{K1} parameter uncertainty.

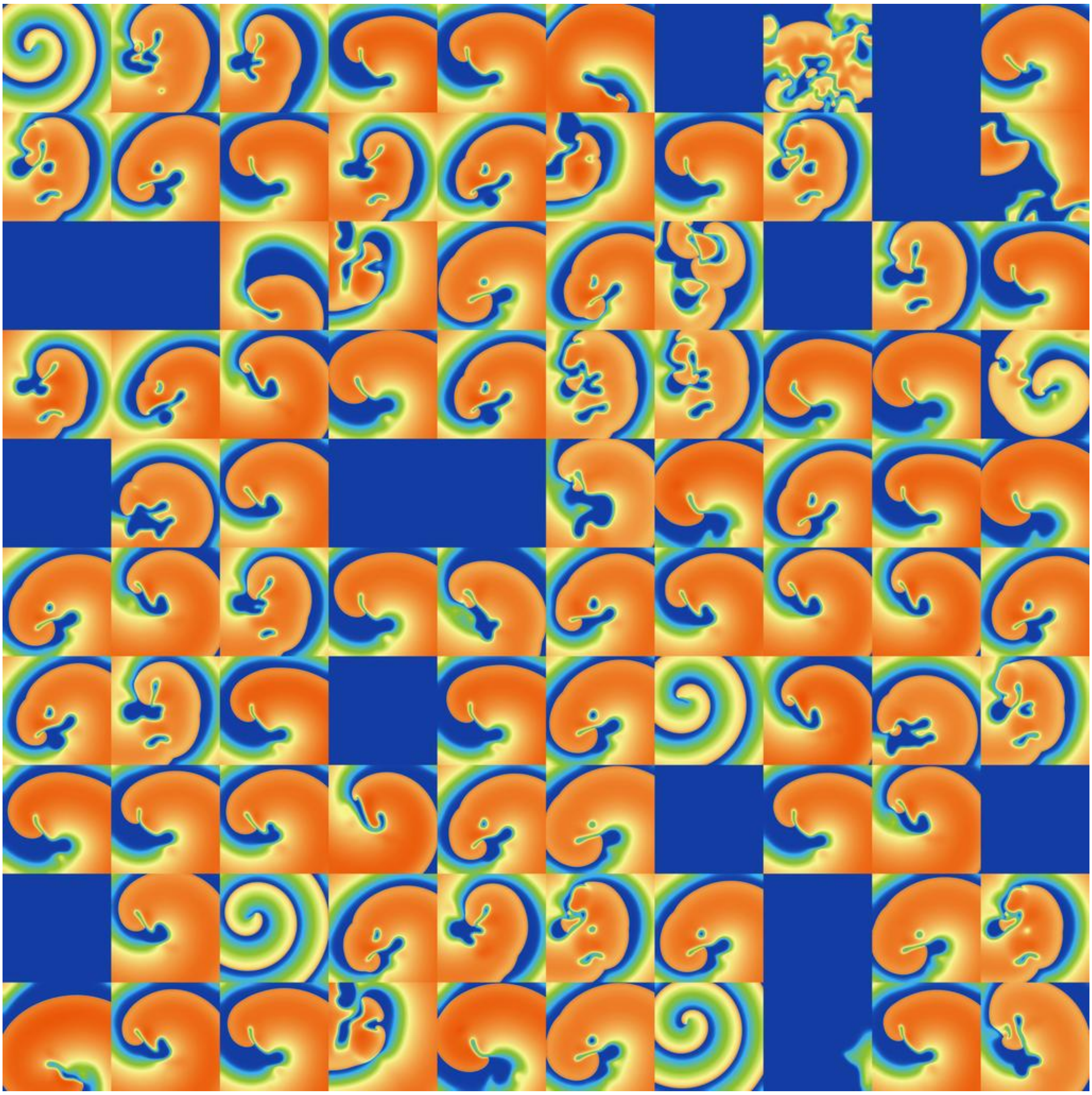

Figure S10. Snapshots of activity at $t = 2000\text{ms}$ for 100 random parameters, under I_{to} parameter uncertainty.

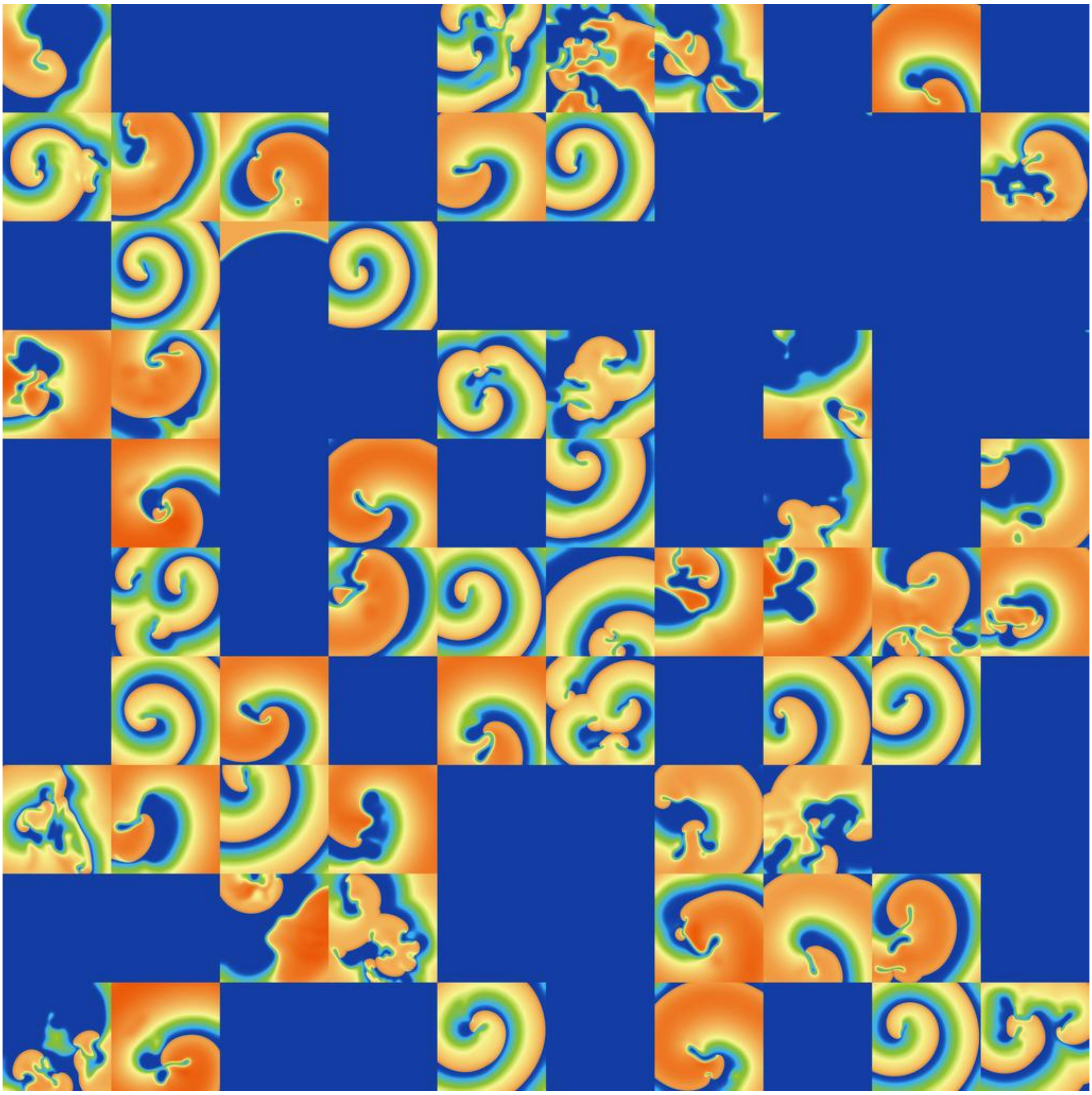

Figure S11. Snapshots of activity at $t = 2000\text{ms}$ for 100 random parameters, under I_{CaL} parameter uncertainty.

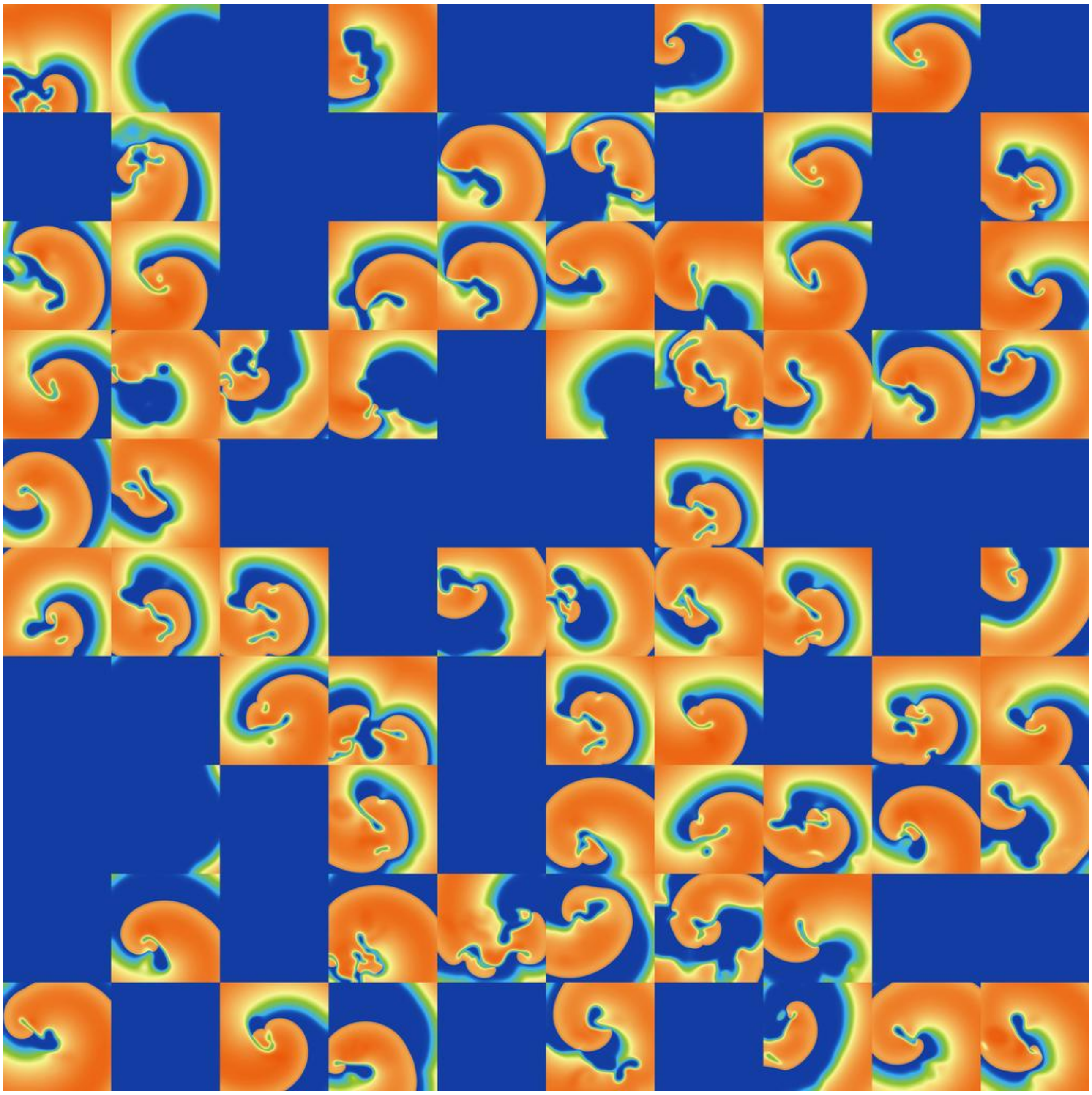

Figure S12. Snapshots of activity at $t = 2000\text{ms}$ for 100 random parameters, under I_{Kr} parameter uncertainty.

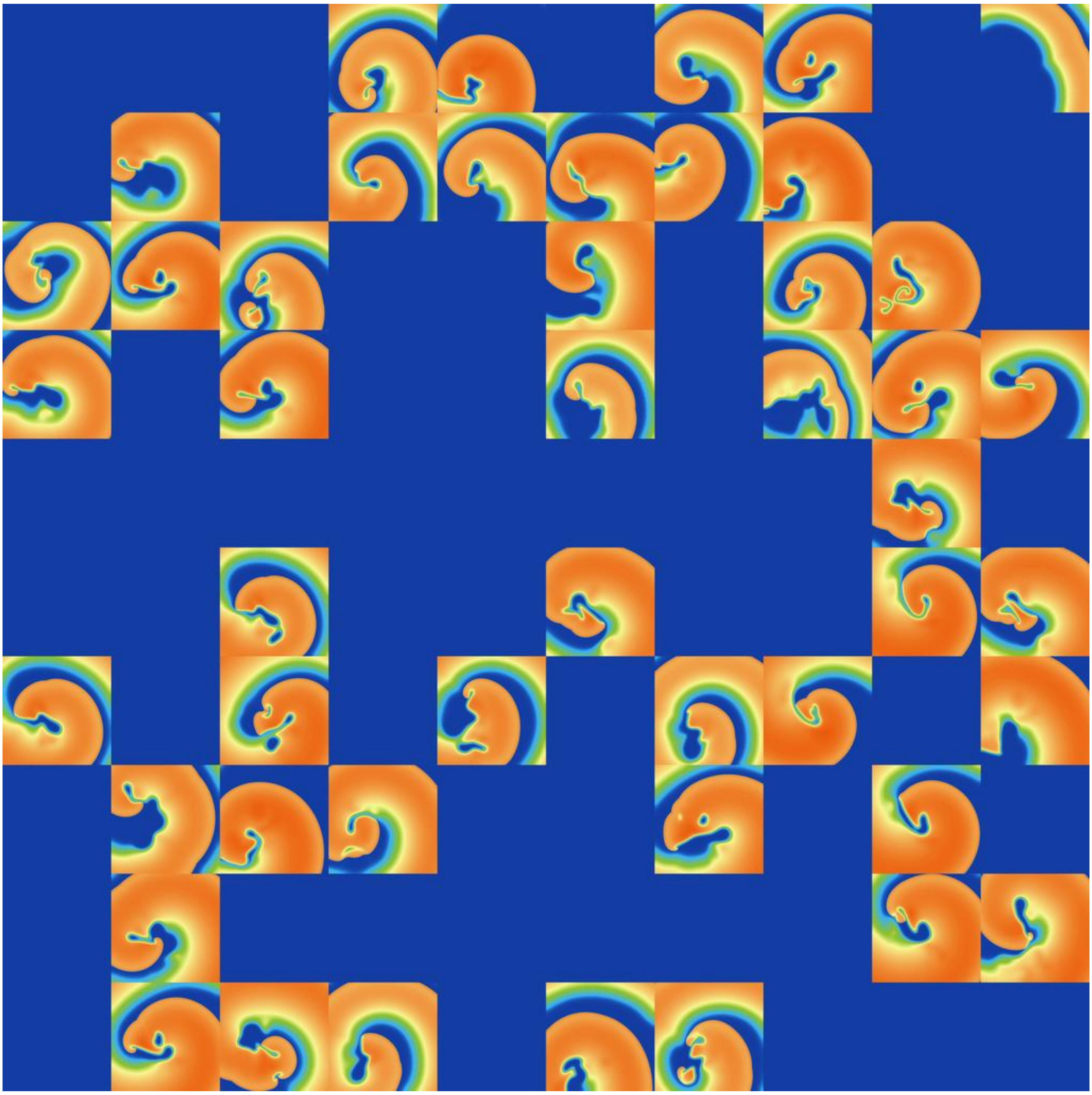

Figure S13. Snapshots of activity at $t = 2000\text{ms}$ for 100 random parameters, under I_{Ks} parameter uncertainty.
